# An Australian chickpea pan‐genome provides insights into genome organization and offers opportunities for enhancing drought adaptation for crop improvement

**DOI:** 10.1111/pbi.70192

**Published:** 2025-06-18

**Authors:** Vanika Garg, Rutwik Barmukh, Yan Huang, Annapurna Chitikineni, Kristy Hobson, Bicheng Yang, Yong Jia, Shengnan Bi, Sukhjiwan Kaur, Muhammad Ahsan Asif, Matthew Hayden, Sally Norton, Darshan L. Sharma, Kadambot H. M. Siddique, Xin Liu, Chengdao Li, Rajeev K. Varshney

**Affiliations:** ^1^ WA State Agricultural Biotechnology Centre, Centre for Crop and Food Innovation, Food Futures Institute Murdoch University Murdoch Western Australia Australia; ^2^ BGI Research Shenzhen China; ^3^ Chickpea Breeding Australia New South Wales Department of Primary Industries and Regional Development, Tamworth Agricultural Institute Tamworth New South Wales Australia; ^4^ BGI‐Australia Herston Queensland Australia; ^5^ Western Crop Genetics Alliance, Food Futures Institute, School of Agriculture Murdoch University Murdoch Western Australia Australia; ^6^ Agriculture Victoria, AgriBio Centre for AgriBioscience Bundoora Victoria Australia; ^7^ Agriculture Victoria Australian Grains Genebank Horsham Victoria Australia; ^8^ Grains Genetic Improvement WA Department of Primary Industries and Regional Development South Perth Western Australia Australia; ^9^ The UWA Institute of Agriculture The University of Western Australia Perth Western Australia Australia

**Keywords:** QTL‐hotspot, graph pan‐genome, flowering time, genetic variation, drought tolerance, haplotype‐based breeding

## Abstract

Chickpea (*Cicer arietinum* L.) is an important legume crop that has been subjected to intensive breeding, resulting in limited genetic diversity. Australia is the world's second largest producer and the leading exporter of chickpea; the genomic architecture of its cultivars remains largely unexplored. This knowledge gap hinders efforts to enhance their genetic potential for production, protection, and stress adaptation. To address this, we generated high‐quality genome assemblies and annotations for 15 leading Australian chickpea cultivars using single‐tube long‐fragment read technology. The pan‐genome analysis identified 34 345 gene families, including 13 986 dispensable families enriched for genes associated with key agronomic traits. Comparative genomic analysis revealed ~2.5 million single‐nucleotide polymorphisms, nearly 200 000 insertions/deletions, and over 280 000 structural variations. These variations were found in key flowering time genes, seed weight‐related genes, and disease resistance genes, providing insights into the genetic diversity underlying these critical traits. Haplotype analysis of key genes within the ‘*QTL‐hotspot*’ region revealed the absence of superior haplotypes in Australian cultivars. Validation using Kompetitive allele‐specific PCR markers confirmed these findings, highlighting the need to introduce beneficial haplotypes from diverse accessions to enhance drought tolerance in Australian chickpea cultivars. The genomic resources generated in this study provide valuable insights into chickpea genetic diversity and offer potential avenues for crop improvement.

## Introduction

Chickpea (*Cicer arietinum* L.) is a major legume crop cultivated in more than 56 countries, primarily on marginal lands, making a substantial contribution to global food and nutritional security. It serves as an essential source of protein, β‐carotene, dietary fibres, and minerals, supporting the nutritional needs of millions, particularly in developing nations. Currently, chickpea cultivation spans ~ 14.10 million hectares worldwide, yielding a total production of about 16.52 million tonnes (FAOSTAT, 2023). Over the past decades, Australia has emerged as the world's second largest producer (after India) and the leading chickpea exporter, with total production exceeding 0.94 million tonnes (FAOSTAT, 2023). Despite this success, chickpea production in Australia faces significant challenges from biotic and abiotic stresses. Therefore, the primary focus of chickpea breeding initiatives in Australia is enhancing environmental adaptability to ensure yield stability and increased productivity.

Advances in sequencing and genotyping technologies have transformed chickpea from a neglected crop to one rich in genomic resources (Roorkiwal *et al*., 2020). The development of the first chickpea reference genome for the cultivated CDC Frontier accession (Varshney *et al*., 2013) has opened avenues for numerous studies focused on profiling genetic variations, including single‐nucleotide polymorphisms (SNPs) and insertions/deletions (InDels), through alignment of short‐read sequencing data (Varshney *et al*., 2019). These genetic markers have been instrumental in identifying key agronomic traits, such as drought tolerance, disease resistance, and yield potential (Barmukh *et al*., 2022a, 2022b; Roorkiwal *et al*., 2020). Their identification and utilization have significantly advanced chickpea breeding programmes by enabling marker‐assisted selection and genomic prediction for trait improvement (Bharadwaj *et al*., 2021).

However, the increasing understanding of genotype variations across different species has highlighted the inadequacy of a single reference genome to fully represent the entire genetic diversity, resulting in significant reference biases (Chapman *et al*., 2022; Khan *et al*., 2020). Identifying structural variations (SVs) based on short‐read sequencing data has proven challenging and unreliable (Sedlazeck *et al*., 2018). Consequently, there is a growing preference for utilizing long‐read sequencing data in plant research for SV identification (Alonge *et al*., 2020). Several studies in plants leveraging high‐quality genome assemblies have successfully elucidated SVs among specific accessions, providing insights into genome evolution, trait diversity, and adaptation to environmental stresses (Hufnagel *et al*., 2020; Ramu *et al*., 2023; Zhou *et al*., 2019). Therefore, to effectively capture the genetic diversity within Australian chickpeas and drive crop improvement, the construction of high‐quality genome assemblies is essential. These assemblies will enable comprehensive cataloguing of genetic variations across diverse genotypes while minimizing reference bias, leading to more accurate assessments of gene presence/absence variations. Currently, such high‐quality genome assemblies are lacking for Australian chickpea cultivars.

The idea of a pan‐genome, typically established through high‐quality genome assemblies of dozens to hundreds of genotypes, aims to encapsulate the entire genomic information of a species (Golicz *et al*., 2016). Pan‐genomes have been constructed for several major crops, including rice (Qin *et al*., 2021), wheat (Jiao *et al*., 2025), barley (Jayakodi *et al*., 2024), and soybean (Liu *et al*., 2020; Torkamaneh *et al*., 2021), among others. For chickpea, a pan‐genome was constructed by integrating short‐read data from 3171 cultivated accessions (both desi and kabuli types) and 28 *Cicer reticulatum* accessions (Varshney *et al*., 2021), while a *Cicer* super‐pan‐genome was developed using genome assemblies from eight wild and two cultivated accessions (Khan *et al*., 2024). These resources have revealed extensive genetic variation, including SVs contributing to stress adaptation. Recent trends in pan‐genome research emphasize capturing the full spectrum of genetic diversity across multiple genotypes, offering a broader perspective than linear reference genomes (He *et al*., 2023; Wang *et al*., 2023b). However, the lack of Australian chickpea pan‐genomes and SV catalogues has limited efforts to identify and harness SVs associated with key traits, such as yield, stress tolerance, and disease resistance.

Haplotype‐based breeding has emerged as a promising strategy for overcoming these challenges. This approach enables the identification and stacking of superior haplotypes to develop improved crop varieties (Barmukh *et al*., 2022a; Singh *et al*., 2024; Sinha *et al*., 2020). In chickpea, superior haplotypes have been identified for yield and yield‐related traits, such as 100‐seed weight, pods per plant, and days to maturity (Varshney *et al*., 2021). Incorporating these haplotypes within a graph‐based pan‐genome framework can enhance breeding precision by facilitating the selection of optimal genetic combinations with improved performance. This strategy could expand the genetic base of Australian chickpeas and accelerate the development of high‐yielding, drought‐tolerant varieties adapted to challenging environmental conditions.

In this study, we generated high‐quality genome assemblies for 15 commercial Australian chickpea cultivars, uncovering a previously uncharacterized reservoir of genetic diversity. We constructed a pan‐genome tailored specifically to Australian chickpea using these genome assemblies. Our analysis identified numerous genetic variations, including hundreds of thousands of SVs, allowing for the discovery of candidate genes associated with key agronomic traits, such as flowering time, disease resistance, and seed weight. Notably, we found that Australian chickpea cultivars analysed lack superior haplotypes of key genes within the drought‐responsive ‘*QTL‐hotspot*’ region, highlighting the need to introduce novel genetic diversity to improve drought adaptation in Australian chickpea cultivars.

## Results

### Genome sequencing and assembly

**Figure 1:**
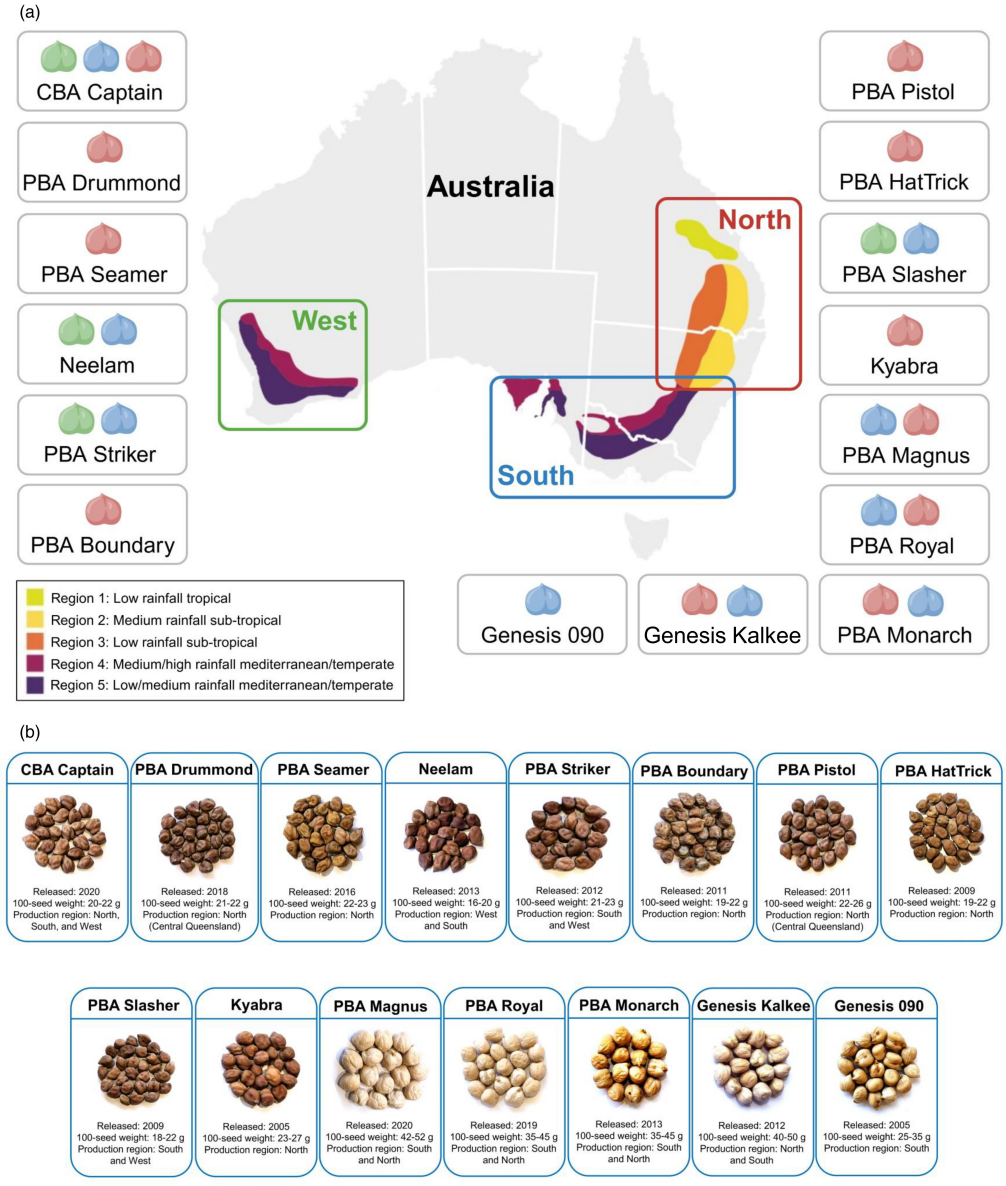
Production regions and characteristics of 15 Australian chickpea cultivars. (a) Map of Australia showing major chickpea‐growing regions categorized into five agroecological zones based on rainfall and climate: Region 1 (low rainfall tropical), Region 2 (medium rainfall sub‐tropical), Region 3 (low rainfall sub‐tropical), Region 4 (medium/high rainfall Mediterranean/temperate), and Region 5 (low/medium rainfall Mediterranean/temperate). The three major chickpea production zones—North, South, and West—are highlighted with their respective adapted chickpea cultivars. Seed symbol colours denote the cultivar's regional adaptation: red for the North, blue for the South, and green for the West, with some cultivars suitable to multiple regions. Source: Pulse Australia and Australian Export Grains Innovation Centre. (b) Photographs of major Australian chickpea cultivars, showing their release year, 100‐seed weight, and primary production region. Desi cultivars (CBA Captain, PBA Drummond, PBA Seamer, Neelam, PBA Striker, PBA Boundary, PBA Pistol, PBA HatTrick, PBA Slasher, and Kyabra) have seed weights ranging from 19 to 29 g per 100 seeds. Kabuli cultivars (PBA Magnus, PBA Royal, PBA Monarch, Genesis Kalkee, and Genesis 090) range from 25 to 45 g per 100 seeds.

This study focused on developing genome assemblies for 15 leading Australian chickpea cultivars released between 2005 and 2020, comprising 10 desi and 5 kabuli types (Figure 1; Table S1). These cultivars exhibit significant agronomic variability, including differences in 100‐seed weight, flowering time, maturity, and disease resistance to *Phytophthora* root rot (Table S1). This genetic diversity provides a strong foundation for capturing genomic variations through genome assemblies, which are essential for understanding and improving agronomic traits in chickpea.

Using single‐tube long‐fragment read (stLFR) technology, we generated 1.75 Tb of sequencing data with an average of 116.95 Gb data (~220.15× coverage) per genotype (Table S2). The raw sequencing data were preprocessed and assembled to generate high‐quality reference‐guided genome assemblies for all cultivars. The contig N50 sizes ranged from 17 Kb to 34.37 Kb, with an average of 23.62 Kb, and scaffold N50 sizes ranged from 53.84 Mb to 63.19 Mb, with an average of 58.60 Mb. The assembled genome size ranged from 406.68 Mb (PBA Royal) to 515.54 Mb (PBA Striker), with an average size of 460.99 Mb. On average, ~93% of each genome assembly was successfully anchored to chromosomes (Table 1).

**Table 1:** Statistics of genome assembly and annotation for 15 Australian chickpea cultivars

Genotype	Type	Scaffold N50 (in Mb)	Assembly size (in Mb)	GC (%)	Chromosome anchoring (%)	Complete BUSCOs (%)	Repeat (%)	Number of genes	Number of transcripts	Genes with annotation (%)
CBA Captain	Desi	61.22	489.85	28.34	92.26	98.90	41.45	32 564	33 623	95.42
PBA Drummond	Desi	59.13	463.08	29.63	93.75	98.60	42.23	32 365	33 434	95.64
PBA Seamer	Desi	57.38	440.91	30.07	94.36	98.80	40.99	30 371	31 453	95.47
Neelam	Desi	63.19	496.85	29.11	93.25	99.00	44.13	32 351	33 281	95.45
PBA Striker	Desi	62.64	515.54	28.70	91.69	99.10	45.16	32 522	33 560	95.43
PBA Boundary	Desi	58.61	456.15	29.38	93.46	98.70	43.39	30 295	31 268	95.42
PBA Pistol	Desi	58.24	463.04	29.78	91.59	98.90	42.49	30 004	30 954	95.52
PBA HatTrick	Desi	57.50	467.96	29.57	90.30	98.90	44.72	30 466	31 399	95.49
PBA Slasher	Desi	61.66	507.12	28.31	90.18	98.90	42.49	32 820	33 871	95.40
Kyabra	Desi	55.70	427.80	30.08	95.20	99.00	41.13	30 148	31 208	95.64
PBA Magnus	Kabuli	57.26	437.02	30.18	95.52	98.70	43.75	29 878	30 895	95.64
PBA Royal	Kabuli	54.24	406.68	29.84	95.81	98.40	39.21	30 503	31 424	95.44
PBA Monarch	Kabuli	53.84	408.50	29.87	95.55	98.50	37.95	30 075	31 039	95.70
Genesis Kalkee	Kabuli	56.69	431.03	30.12	95.69	98.80	42.25	30 176	31 149	93.26
Genesis 090	Kabuli	61.74	503.27	28.62	92.50	98.90	44.87	31 308	32 298	95.30

The GC content ranged from 28.31% in PBA Slasher to 30.18% in PBA Magnus (Figure S1). The repeat content of the assemblies ranged from 37.95% (PBA Monarch) to 45.16% (PBA Striker). Among the repetitive sequences, long terminal repeat (LTR) retrotransposons were the most abundant, consistent with previous studies (Garg *et al*., 2022). By integrating homology searches, *ab initio* prediction, and transcript evidence, we predicted an average of 31 056 gene models. The mRNA, CDS, intron, and exon lengths were similar across all chickpea cultivars (Figure S2). The majority of predicted genes (95.35% average) in each of the genome assemblies were assigned functional annotations using different publicly available databases (Table 1). We also identified an average of 119 miRNA, 483 rRNA, 1097 tRNA, and 583 snoRNA genes from these genome assemblies (Table S3). Further, the genome assemblies and predicted gene models were evaluated for completeness using Benchmarking Universal Single‐Copy Orthologs (BUSCO; Manni *et al*., 2021). On average, 98.81% of the 1440 core Embryophyta genes were identified in all genome assemblies, confirming their high‐quality and completeness (Table 1).

### Core and dispensable genomes

**Figure 2:**
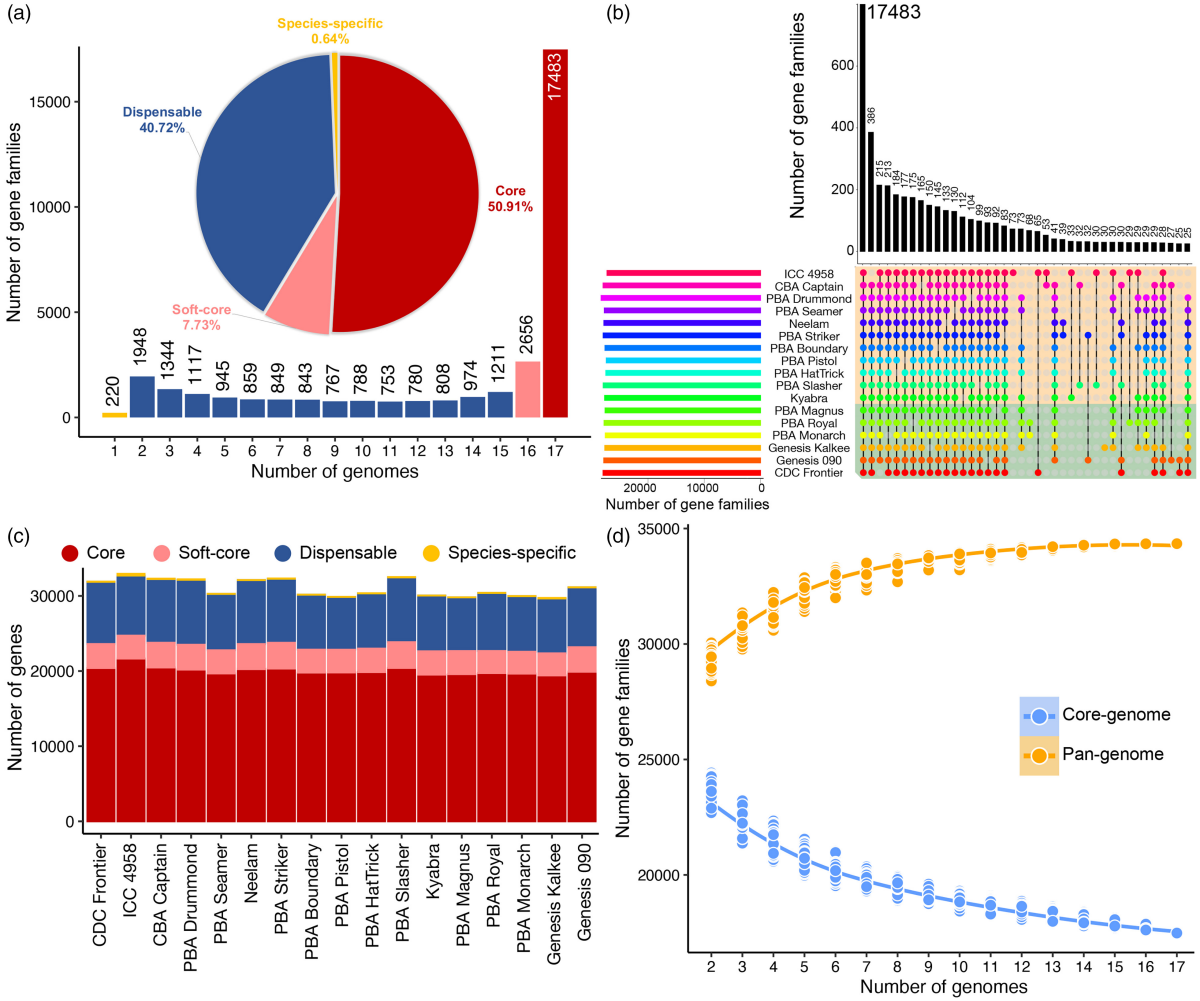
Pan‐genome analysis of the Australian cultivars. (a) Compositions of the pan‐genome and individual genomes. The histogram denotes the number of gene families across the 17 genomes at different frequencies, while the pie chart displays the proportion of gene families identified. Yellow bars highlight species‐specific gene families in each of the genomes. (b) An UpSet plot illustrating the number of gene families shared among different chickpea cultivars. Horizontal bars represent the number of gene families per genotype. (c) Gene family composition across all chickpea cultivars. (d) Modelling curve for the core and pan‐genome. Simulations depict an increasing pan‐genome size and decreasing in core genome size as more cultivars are included. Each simulation was performed for 1000 times per genotype count. Solid black lines indicate median gene numbers.

To investigate the genetic diversity in chickpea, we performed comprehensive pan‐genome analysis using 15 newly assembled genomes and the ICC 4958 and CDC Frontier reference genomes. Ortholog investigation classified all genes from the 17 chickpea genomes into 34 345 gene families (Table S4). These gene families were classified into four categories based on their distribution: (i) 17 483 (50.91%) families present in all 17 cultivars were designated as core gene families, representing conserved genes for the species; (ii) 2656 (7.73%) gene families, present in at least 16 cultivars (>90% of genomes), were classified as softcore gene families; (iii) 13 986 (40.72%) gene families were found in 2–15 cultivars and categorized as dispensable gene families, reflecting substantial genetic variation among cultivars; and (iv) 220 (0.64%) gene families exclusive to a single genotype were termed private gene families (Figure 2a). Additionally, 3561 singleton genes were identified, representing a reservoir of unique alleles in cultivated chickpea. Notably, 41 gene families were found exclusively in Australian cultivars (Figure 2b). Dispensable and genotype‐specific gene families accounted for 41.36% of the total gene families and represented 26.39% of all genes (Figure 2c).

Gene ontology (GO) enrichment analysis provided insights into the biological significance of these gene families. Core gene families were predominantly enriched for genes involved in fundamental biological processes, such as transcriptional regulation, lipid biosynthesis, DNA replication, and protein folding, which are critical for the maintenance of basic cellular functions (Figure S3). In contrast, dispensable gene families were associated with stress adaptation, including defence response, DNA repair, telomere maintenance, and cell wall modification. Furthermore, private gene families and singletons were mainly enriched for specialized functions, including bacteriocin transport, adenine DNA methylation, DNA‐mediated transposition, extracellular polysaccharide biosynthesis, and peptidoglycan metabolism. These findings are consistent with previous pan‐genome studies in chickpea (Khan *et al*., 2024).

The gene family modelling analysis revealed that the total number of gene families increased with the inclusion of additional genomes, reaching a near plateau at ~11 genomes (Figure 2d). This trend suggests that these 17 cultivars capture most of the genetic diversity in cultivated chickpea. Importantly, this plateau indicates that sequencing additional cultivated cultivars is unlikely to reveal significant novel gene families, emphasizing the completeness of this dataset in representing the genomic diversity of cultivated chickpea.

### Variation catalogue for Australian chickpea

#### SNPs and small InDels

**Figure 3:**
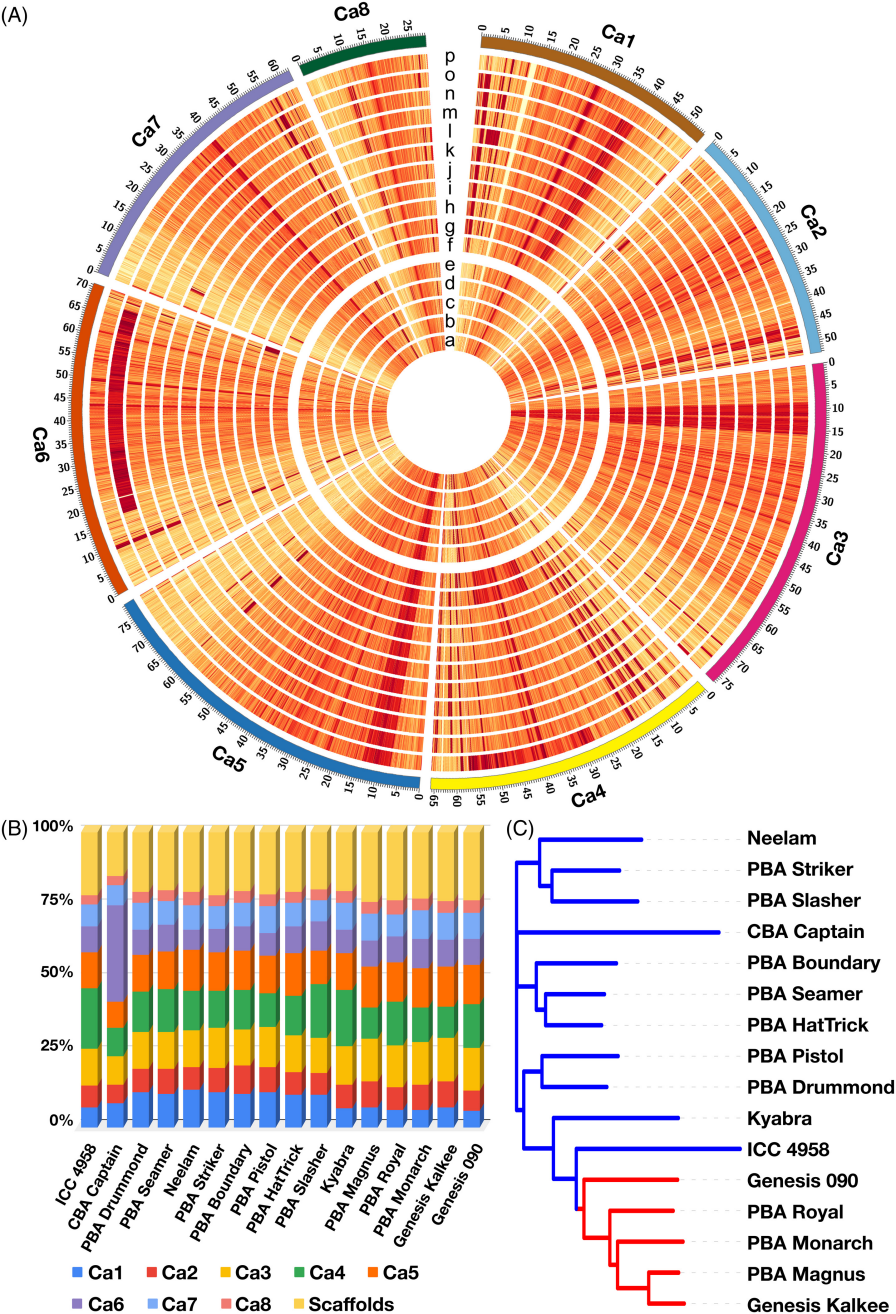
Genome‐wide distribution of SNPs in 15 Australian chickpea cultivars. (A) Circos plot visualizing SNP distribution across Australian chickpea cultivars. The five inner tracks represent kabuli cultivars (a–e) ordered by their release year: (a) Genesis 090; (b) Genesis Kalkee; (c) PBA Monarch; (d) PBA Royal; (e) PBA Magnus. The 11 outer tracks represent desi cultivars (f–o) ordered by release year: (f) Kyabra; (g) PBA Slasher; (h) PBA HatTrick; (i) PBA Pistol; (j) PBA Boundary; (k) PBA Striker; (l) Neelam; (m) PBA Seamer; (n) PBA Drummond; (o) CBA Captain. The outermost track (p) denotes ICC 4958. SNP density calculated over 50 kb windows is indicated by a colour scale, with yellow and red denoting low and high density, respectively. (B) Stacked bar plot showing the chromosome‐wise distribution of SNPs for each genotype. (C) Phylogenetic tree constructed using genome‐wide SNP data from the Australian cultivars and ICC 4958. Red and blue branches indicate kabuli and desi cultivars, respectively.

To assess sequence diversity among the 15 Australian chickpea cultivars, we aligned their raw genomic data to the CDC Frontier reference genome (Garg *et al*., 2022). This analysis identified 2 473 162 non‐redundant (unique genomic locus) SNPs and 213 633 InDels (Tables S5, S6; Figure 3; Figure S4). Across the eight chromosomes, the highest number of SNPs was detected on Ca6 (590 013 [8331 per MB]; 23.86%), followed by Ca4 (393 226 [5976 per Mb]; 15.90%), while Ca8 had the lowest count (75 911 [2646 per Mb]; 3.07%) (Figure 3A; Table S5). SNP count per cultivar ranged from 839 464 (1583 per Mb; Genesis Kalkee) to 1 538 962 (2902 per Mb; CBA Captain). Interestingly, while previous studies identified Ca4 as harbouring the highest number of SNPs, our results showed that Ca6 had the most SNPs, largely due to the unusually high SNP density in the CBA Captain genotype on Ca6 (Figure 3B). CBA Captain had the highest number of SNPs among desi cultivars, while Genesis 090 had the most among kabuli cultivars. Phylogenetic analysis of these SNPs classified the cultivars into two distinct groups, one containing Neelam, PBA Striker, PBA Slasher, CBA Captain, PBA Boundary, PBA Seamer, PBA HatTrick, PBA Pistol, PBA Drummond, Kyabra, and ICC 4958, and another comprising Genesis 090, PBA Royal, PBA Monarch, PBA Magnus, and Genesis Kalkee. These clusters aligned with the market class, with all desi cultivars forming one group and all kabuli cultivars clustering separately (Figure 3C).

The distribution of InDels (<50 bp) across chromosomes was similarly uneven. Ca4 contained the highest number of InDels (42 670 [648 per Mb]; 19.97%), whereas Ca8 had the lowest (10 175 [355 per Mb]; 4.76%) (Table S6). InDel abundance varied significantly among cultivars, with PBA Magnus showing the fewest InDels (69 674 [131 per Mb]) and CBA Captain the most (122 599 [231 per Mb]). On average, desi cultivars had a higher InDel count (104 282 [197 per Mb]) than kabuli cultivars (72 963 [138 per Mb]), likely reflecting the kabuli origin of the reference genome.

Functional annotation of these genome‐wide variations revealed that most SNPs (91.02%) were located in intergenic regions, with intronic regions accounting for 6.42% and exonic regions for 2.43% (Table S7). Within exonic SNPs, 35.18% were missense mutations, 0.96% were nonsense, 33.40% were synonymous, and 30.46% were UTR variants. Notably, the CBA Captain had the highest number of missense SNPs (7113), highlighting its potential contribution to functional diversity.

The variation in SNP and InDel abundance across chromosomes and cultivars underscores the genetic diversity within Australian chickpea. Missense mutations and InDels can alter gene function, potentially influencing traits related to growth, development, and resilience to biotic and abiotic stresses. The distinct diversity levels across cultivars suggest possible adaptive traits. Future studies on functional characterization will be critical to uncover their utility for breeding applications.

#### Genome organization of Australian chickpeas

**Figure 4:**
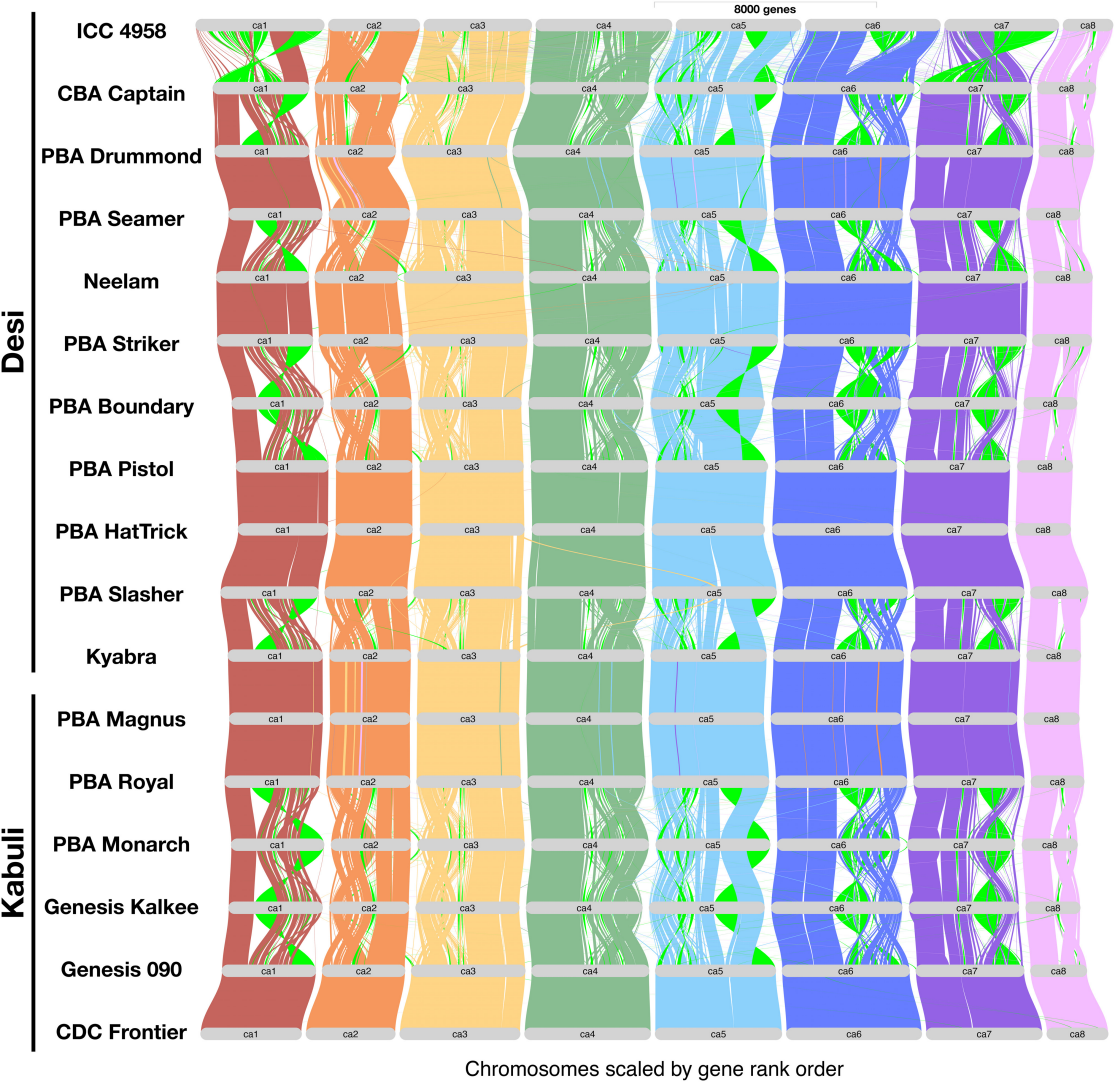
Whole‐genome synteny alignment of Australian chickpea cultivars, CDC Frontier and ICC 4958. The braids represent the eight chickpea chromosomes, with ribbons indicating the syntenic relationship between the different chromosomes of the cultivars. Ribbon colours correspond to CDC Frontier chromosome assignment, where green ribbons highlight inversions. The plot illustrates major structural rearrangements among chickpea cultivars.

We conducted a gene‐based synteny analysis to investigate genomic differences among Australian chickpea cultivars. Structural comparisons of genome assemblies from 10 desi and five kabuli cultivars were performed alongside the high‐quality reference genomes of ICC 4958 (Chattopadhyay and Francis, 2021) and CDC Frontier (Garg *et al*., 2022). Synteny plots illustrated chromosomal rearrangements and inversions depicted by green lines (Figure 4). The analysis revealed a high degree of gene collinearity across all eight chromosomes (Ca1–Ca8), indicating a largely conserved genomic structure among cultivars. However, significant genome reorganization was observed in Ca1, Ca5, Ca6, and Ca7, with notable SVs in desi cultivars (ICC 4958, CBA Captain, PBA Drummond, PBA Seamer, PBA Boundary, and Kyabra) compared with kabuli cultivars (PBA Monarch, Genesis Kalkee, and Genesis 090) (Figure 4). These variations, characterized by distinct syntenic disruptions and translocations, suggest higher chromosomal rearrangement frequencies in desi types compared with kabuli types, likely reflecting genomic evolution and adaptation to diverse environmental conditions.

#### Structural variations

Structural variations (SVs), including large deletions, insertions, duplications, and chromosomal rearrangements, are a major source of genetic diversity and can influence agronomic traits such as flowering time and disease resistance (Alonge *et al*., 2020; Yuan *et al*., 2021). However, identifying SVs using short‐read sequencing is challenging. To overcome this limitation, this study utilized stLFR technology, which provides barcoded reads that retain long‐range linkage information, thereby improving the detection and resolution of large and complex SVs. A total of 280 195 SVs overlapping 12 440 unique genes were identified across the 15 Australian cultivars and ICC 4958, including 113 357 insertions, 164 157 deletions, 291 inversions, 259 duplications, and 2131 translocations (Table S8). Among the chromosomes, Ca5 (47 808) had the most SVs, followed by Ca3 (47 122) and Ca6 (39 964). The largest inversion (45.82 Mb) was detected on Ca6 in PBA Royal. The largest duplication (95.62 Kb) was identified on Ca7 in PBA Slasher and contained the gene encoding an E3 ubiquitin–protein ligase MBR2‐like protein (*Ca_v2.0_21798*), which may be involved in protein degradation and abiotic stress tolerance (Chai *et al*., 2024). Another duplication (72.35 Kb) on Ca3 in Genesis 090 contained genes encoding an L‐type lectin‐domain‐containing receptor kinase IX.1‐like protein and low‐molecular‐weight cysteine‐rich protein, which plays a role in plant defence (Sun *et al*., 2020). The largest deletion (118.86 Kb) was found in Genesis 090, leading to the loss of six genes, including those encoding E3 ubiquitin–protein ligase RLIM‐like proteins and the anaphase‐promoting complex subunit 11 RING‐H2 finger protein, both of which are involved in transcriptional regulation and cell cycle control (Mazzucotelli *et al*., 2006). Additionally, a large intergenic deletion (113.72 Kb) on Ca5 was identified in five Australian cultivars (Genesis Kalkee, PBA Magnus, PBA Royal, Kyabra, and PBA Seamer). Notably, a 5.9 Kb deletion on Ca6, present in all Australian cultivars, harboured the gene encoding a general transcription factor 3C polypeptide 3‐like protein (*Ca_v2.0_17667*). Another deletion (4.48 Kb) on Ca5, also conserved across all Australian cultivars, resulted in a bidirectional gene fusion. Furthermore, an intronic insertion of 323 bp was identified on Ca5 within a gene encoding alpha‐N‐acetylglucosaminidase. Two deletions were observed specifically in kabuli cultivars but absent in desi cultivars: 121 bp deletion on Ca7 (affecting insulin‐degrading enzyme‐like gene, *Ca_v2.0_22576*) and 321 bp deletion on Ca3 (affecting receptor‐like protein kinase gene, *Ca_v2.0_08091*). Furthermore, a 2979 bp deletion on Ca1, specific to kabuli cultivars, was observed in a gene encoding for glutamate receptor 2.7‐like (*Ca_v2.0_00379*), which may affect signalling and nitrogen uptake (Dey *et al*., 2025). These examples were selected to represent the diversity, recurrence, and functional relevance of SVs across cultivars. These findings highlight the extensive genetic diversity and SVs in Australian chickpea cultivars, providing insights into their genetic diversity and the influence of identified SVs on potential agronomic traits.

With CDC Frontier serving as the backbone, SVs from 15 Australian cultivars and ICC 4958 were integrated into a variation graph. This chickpea graph genome spans 986 799 923 bp, containing 31 063 865 nodes and 31 229 144 edges. This resource provides a superior reference for cultivated chickpea, capturing the full spectrum of structural variations and offering a superior platform for accurate genotyping and trait association studies compared to a single reference genome.

#### Variations in flowering time‐related genes

Flowering time variation is crucial for chickpea adaptation to diverse agroclimatic environments and optimizing yield (Berger *et al*., 2004). Developing new chickpea varieties suited to different growing regions and improving existing ones relies on identifying and incorporating allelic variations at key genes that regulate flowering time. This study identified 263 genes associated with flowering time through sequence similarity with genes from other plant species, with their variations analysed using pan‐genome analysis (Table S9). Across ICC 4958 and 15 Australian chickpea cultivars, 2980 variations were detected within 201 flowering time‐related genes. Specifically, 2598 SNPs were found in 181 unique genes, with an average of ~14.35 SNPs per gene. Ca6 contained the most SNPs linked to flowering time‐related genes, while Ca2 had the fewest. Similarly, 382 InDels were identified in 118 unique genes, averaging ~3.24 InDels per gene. Ca4 had the highest number of InDels, whereas Ca5 and Ca8 had the lowest.

Further analysis identified specific flowering time‐related genes with SNPs or InDels in their coding sequences, which were predicted to significantly affect protein function. For example, the flowering time‐related gene *Ca_v2.0_07034* on Ca3, encoding a FLOWERING LOCUS T (FT)‐like protein, plays a key role in initiating flowering at the shoot apical meristem (Takagi *et al*., 2023). This gene contained a missense variant (Leu90Trp), with the allele ‘G/G’ associated with early‐flowering cultivars (e.g., PBA Striker and PBA Pistol), while the allele ‘T/T’ was linked to mid‐ and mid‐late flowering cultivars such as CBA Captain, PBA Drummond, PBA HatTrick, Genesis 090, and Genesis Kalkee. Given that kabuli chickpea cultivars generally require a longer flowering duration to better adapt to varying environmental conditions (Purushothaman *et al*., 2014), these genetic variations suggest potential differences in flowering regulation between desi and kabuli types.

Another key flowering time‐related gene, *Ca_v2.0_00299*, encodes a mediator of RNA polymerase II transcription subunit 16 (MED16)‐like, which functions as part of the Mediator complex and regulates RNA polymerase II‐dependent transcription. This gene, potentially linked to flowering time in chickpea (Perez‐Rial *et al*., 2024), contained nine missense SNPs and three splice region and intron SNPs, distinguishing the ten Australian desi cultivars from five kabuli cultivars. Additionally, the *Ca_v2.0_11676* gene, which encodes thermospermine synthase ACAULIS5 (ACL5), a regulator of organ elongation and meristematic activity during the reproductive phase in *Arabidopsis* (Hanzawa *et al*., 1997), was affected by a splice region variant and an intronic InDel, further differentiating the desi cultivars from the kabuli.

#### Variations in resistance genes

Chickpea is affected by a range of devastating biotic stresses, including Ascochyta blight, Phytophthora root rot, Fusarium wilt, and Botrytis grey mould, among others (Choudhary *et al*., 2023). A comprehensive catalogue of resistance (R) genes provides a valuable resource for breeding to improve disease resistance. Various resistance gene analogs (RGA) conferred a significant portion of chickpea disease resistance, including NBS‐encoding proteins, with the CNL class being the most abundant, followed by NL and TX. Across the chickpea cultivars, the number of NBS‐encoding genes ranged from 114 in PBA Striker and PBA Hatrick to 132 in Kyabra. Variation analysis across these cultivars revealed 1039 variations affecting 93 R genes (Table S10). Specifically, 924 SNPs were identified in 84 unique R genes, averaging ~11 SNPs per gene. Additionally, 115 InDels were found in 48 unique R genes, with an average of ~2.4 InDels per gene. Ca6 had the most SNPs associated with R genes, while Ca5 and Ca7 harboured the most InDels. In contrast, Ca2 and Ca8 had the fewest SNPs and InDels, respectively, linked to R genes.

Among the identified R genes, *Ca_v2.0_14823*, encoding calcium‐transporting ATPase 1, is a key regulator of intracellular Ca^2+^ concentrations and supports cell signalling responses (Ding *et al*., 2011). This gene contained a missense SNP variant (Leu624Phe), where the ‘T/T’ allele was associated with highly susceptible cultivars to Ascochyta blight (Pathogen Group 2 – North), including PBA Drummond and PBA Pistol. In contrast, the ‘C/C’ allele was linked to cultivars classified as moderately susceptible or susceptible to the disease. Another gene, *Ca_v2.0_03169*, annotated as an uncharacterized protein, contained three missense variants (Val187Leu, Arg220His, and Cys229Tyr) that differentiated highly susceptible cultivars from those that were moderately susceptible or susceptible to Ascochyta blight (Pathogen Group 2 – North). Similarly, *Ca_v2.0_23927*, also an uncharacterized protein, carried a missense variant (Ala1429Thr) that distinguished moderately susceptible cultivars (e.g., PBA Royal and Genesis 090) from those classified as susceptible and very susceptible to Ascochyta blight (Pathogen Group 1 – South). Interestingly, the *Ca_v2.0_22430* gene, which encodes an NB‐ARC domain disease resistance protein containing a functional ATPase domain, plays a regulatory role in R protein activity through its nucleotide‐binding state (van Ooijen *et al*., 2008). This gene was affected by a frameshift InDel variant, which distinguished the ten Australian desi cultivars (CA/CA) from the five kabuli cultivars (CAA/CAA).

#### Variations in seed weight‐related genes

Seed weight and size are critical traits in chickpea, influencing yield and market value. Larger seeds are particularly desirable in global markets due to their higher trade value. Although chickpea cultivars exhibit significant phenotypic variation in seed weight (Varshney *et al*., 2021), this potential has not been exploited fully in breeding programmes. A major limitation is the incomplete understanding of genetic variations underlying key candidate genes controlling this trait. We identified 43 genes associated with seed weight and size using sequence similarity with homologous genes from other plant species. Pan‐genome analysis revealed 787 genetic variations, including SNPs and InDels, across ICC 4958 and 15 Australian chickpea cultivars (Table S11). Specifically, 666 SNPs were detected in 41 unique genes, with an average of ~16.24 SNPs per gene, while 121 InDels were identified in 34 unique genes, averaging ~3.56 InDels per gene. Ca4 had the most SNPs and InDels associated with seed weight‐related genes, whereas Ca5 had the fewest.

We further identified specific seed weight‐related genes containing SNPs or InDels that were predicted to significantly affect protein function. For example, the *Ca_v2.0_22437* gene on Ca7, which encodes Growth‐Regulating Factor 5 (GRF5), functions as a transcription activator. GRF5, in conjunction with GIF1, regulates seed weight/size and shape by promoting or maintaining cell proliferation activity (Ge *et al*., 2016; Horiguchi *et al*., 2005). This gene harboured two missense variants (Thr136Asn and Asn286Thr) and one 5′ UTR InDel variant that distinguished desi cultivars (Neelam, PBA Slasher, PBA HatTrick, PBA Boundary, CBA Captain, PBA Drummond, PBA Striker, PBA Seamer, PBA Pistol, and Kyabra) with low seed weight, from kabuli cultivars (Genesis 090, PBA Monarch, PBA Royal, Genesis Kalkee, and PBA Magnus), with high seed weight. Additionally, a gene from the TIFY family, which encodes plant‐specific transcription factors characterized by a highly conserved TIFY domain, plays a key role in regulating seed weight, organ development, and stress tolerance (Bai *et al*., 2011; Barmukh *et al*., 2022b; Liu *et al*., 2022b). The *Ca_v2.0_11263* gene on Ca4, encoding the TIFY 6A protein, contained four missense variants (Ser94Asn, Val109Ala, Ser165Pro, and Asn180Tyr) and one 5′ UTR InDel variant. These genetic variations effectively differentiated all desi cultivars from kabuli cultivars based on seed weight. The variations identified in key seed weight‐related genes, which distinguish small‐ and large‐seeded desi and kabuli cultivars, provide valuable opportunities for functional validation and targeted deployment in breeding programmes to improve seed weight.

#### Variations underlying the ‘QTL‐hotspot’ region

A genomic region on Ca4, known as the ‘*QTL‐hotspot*’ region, has been associated with key drought‐adaptive traits in chickpea (Barmukh *et al*., 2022a; Varshney *et al*., 2014). In this study, we examined genetic variations within 26 genes located in the ~300‐kb ‘*QTL‐hotspot*’ region, defined by the bin markers bin_4_13239546 and bin_4_13547009 (Kale *et al*., 2015). These bin markers indicate recombination‐suppressed genomic intervals identified using a sliding window approach, where adjacent regions without recombination events across the recombinant inbred line population were grouped and treated as single markers for high‐resolution genetic mapping. We identified 700 polymorphic variants, including 534 SNPs and 166 InDels, in this region across 15 Australian chickpea cultivars and ICC 4958 (Table S12). Of these, 42 were within genic regions, comprising 17 intronic and 25 exonic variants. More specifically, 11 were missense mutations, 14 were synonymous mutations, 16 were intronic, and one was a splice donor variant. Additionally, 658 variations were detected in intergenic regions. When examining SNP distribution, most were located in intergenic regions (93.45%) and were more prevalent in exons (4.68%) than in introns (1.87%). The exonic variations included two missense SNPs in the *CaTIFY4b* gene, which regulates key drought‐adaptive traits in chickpea, such as early vigour, root system architecture, and seed weight (Bai *et al*., 2011; Barmukh *et al*., 2022b; Liu *et al*., 2022b). Additionally, four missense mutations were identified in the *vicilin* gene, a key seed storage protein that functions as a carbon and nitrogen reserve, providing energy during germination (Bose *et al*., 2024). Furthermore, five missense variants were found in four additional genes, encoding a stem‐specific protein TSJT1, an emp24 gp25L p24 family protein, an epidermal patterning factor 5, and homocysteine S‐methyltransferase.

### Haplotype analysis of candidate genes underlying the ‘*QTL‐hotspot*’ region

**Figure 5:**
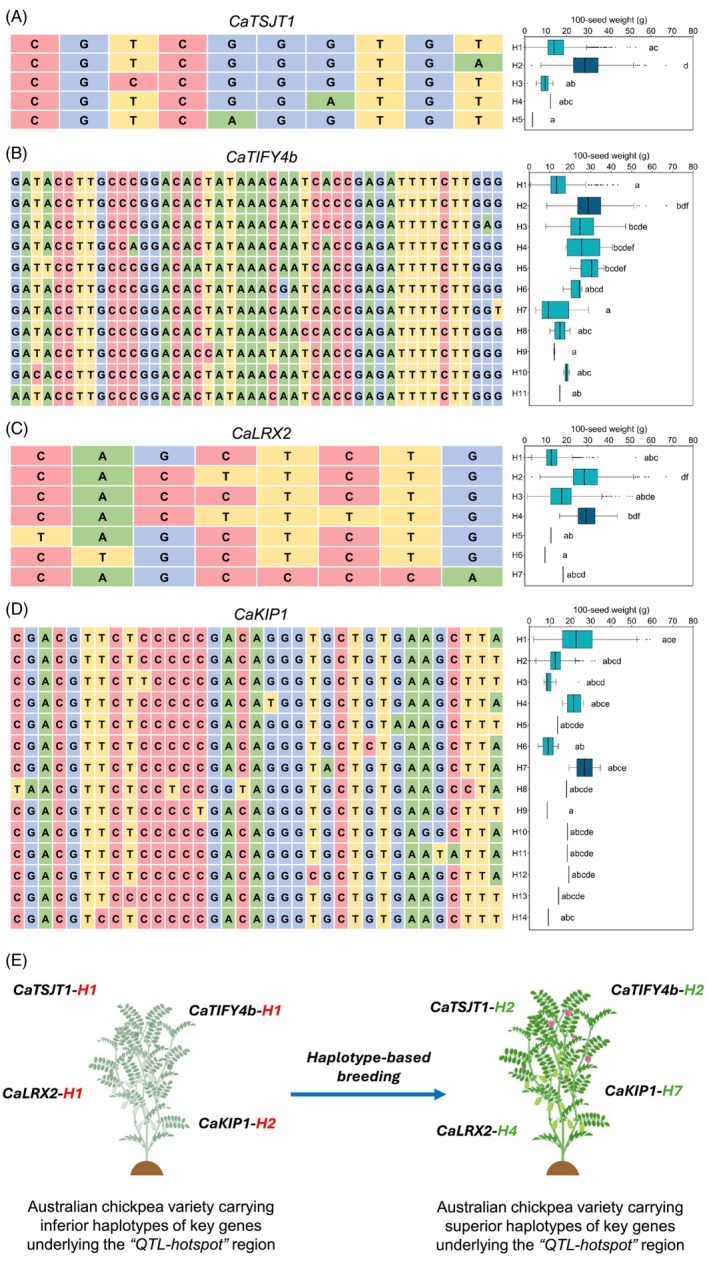
Haplotype analysis of key genes within the ‘*QTL‐hotspot*’ region. (A–D) Haplotype analysis of four key genes (*CaTSJT1*, *CaTIFY4b*, *CaLRX2*, and *CaKIP1*) associated with 100‐seed weight in chickpea. Each panel shows haplotype variations with single‐nucleotide polymorphisms (SNPs) colour‐coded for clarity. Box plots display the distribution of 100‐seed weight (g) across different haplotypes, with statistically significant differences (*P*‐value <0.05) denoted by letters. (A) *CaTSJT1*: Four haplotypes (H1–H4) were identified, with *CaTSJT1‐H2* being the superior haplotype. (B) *CaTIFY4b*: About 11 haplotypes (H1–H11) were detected, with *CaTIFY4b‐H2* exhibiting the highest seed weight. (C) *CaLRX2*: Seven haplotypes (H1–H7) were identified, with *CaLRX2‐H4* being the superior haplotype. (D) *CaKIP1*: About 14 haplotypes (H1–H14) were observed, with *CaKIP1‐H7* showing the highest seed weight. Dark cyan blue indicates the superior haplotype, while bright cyan represents other haplotypes. (E) Schematic representation of haplotype‐based breeding for improving Australian chickpea cultivars. The left diagram depicts chickpea variety carrying inferior haplotypes for all four genes, while the right diagram illustrates a high‐yielding drought‐adaptive variety incorporating superior haplotypes (*CaTSJT1‐H2*, *CaTIFY4b‐H2*, *CaLRX2‐H4*, and *CaKIP1‐H7*).

Haplotype analysis of 26 genes within the ‘*QTL‐hotspot*’ region was conducted using whole‐genome sequencing data from 3171 cultivated chickpea accessions (Varshney *et al*., 2021). Of these, 22 genes exhibited multiple haplotypes (two or more) (Table S13). The number of haplotypes varied significantly, with genes *CaHSFA3* (encoding heat shock transcription factor A3 and lisH domain) and *CaKIAA1468* (encoding HEAT repeat containing protein KIAA1468) displaying the fewest haplotypes (two each). In contrast, gene *CaKIP1* (encoding kinase interacting (KIP1‐like) family protein) exhibited the most haplotypes (14). Haplotype‐phenotype analysis identified superior haplotypes for key genes associated with 100‐seed weight (Figure 5A–D). Notably, *CaTIFY4b* exhibited significant variation in 100‐seed weight among 11 haplotypes, which were classified into different groups using Duncan's test (Figure 5B; Table S14). Accessions carrying the superior haplotype (*CaTIFY4b‐H2*) recorded the highest 100‐seed weight (mean = 29.84 g), while those with the inferior haplotype (*CaTIFY4b‐H1*) had the lowest weight (mean = 14.66 g). Similarly, *CaTSJT1* (encoding stem‐specific protein TSJT1) had five haplotypes, with the superior haplotype (*CaTSJT1‐H2*) associated with significantly higher 100‐seed weight than the other four haplotypes (Figure 5A; Table S15). *CaLRX2* (encoding LRR extensin 2) and *CaKIP1* also exhibited superior haplotypes, identified as *CaLRX2‐H4* and *CaKIP1‐H7*, respectively (Figure 5C,D; Tables S16, S17). The haplotype information for the aforementioned 22 genes within the ‘*QTL‐hotspot*’ region (Varshney *et al*., 2021) was applied in the present study to assess haplotype diversity among Australian chickpea cultivars.

Building on this, we analysed the haplotype combinations in 15 Australian chickpea cultivars to determine whether they carried superior haplotypes for key genes within the ‘*QTL‐hotspot*’ region. For *CaTIFY4b*, ICC 4958 (donor genotype for the ‘*QTL‐hotspot*’ region) carried the superior haplotype *CaTIFY4b‐H2* (associated with high 100‐seed weight), but none of the Australian cultivars had this haplotype. Among the Australian cultivars, all desi cultivars, Genesis 090 and PBA Royal, carried the inferior haplotype (*CaTIFY4b‐H1*), while PBA Magnus, PBA Monarch, and Genesis Kalkee carried *CaTIFY4b‐H3* (Table S14). For *CaTSJT1*, the superior haplotype *CaTSJT1‐H2* was absent in all Australian desi cultivars, which instead carried *CaTSJT1‐H1* (Table S15). For *CaLRX2*, the superior haplotype *CaLRX2‐H4* was not present in any Australian variety, which instead carried *CaLRX2‐H1*, *CaLRX2‐H2*, or *CaLRX2‐H3* (Table S16). For *CaKIP1*, the superior haplotype *CaKIP1‐H7* was absent in all Australian cultivars, which instead carried *CaKIP1‐H2* or *CaKIP1‐H1* (Table S17). These findings highlight the absence of superior haplotypes for key genes within the ‘*QTL‐hotspot*’ region in most leading Australian chickpea cultivars, underscoring the need for targeted breeding efforts to introduce beneficial alleles that could enhance seed weight and drought adaptation (Figure 5E).

### Validation of superior haplotypes in the ‘*QTL‐hotspot*’ region

**Figure 6:**
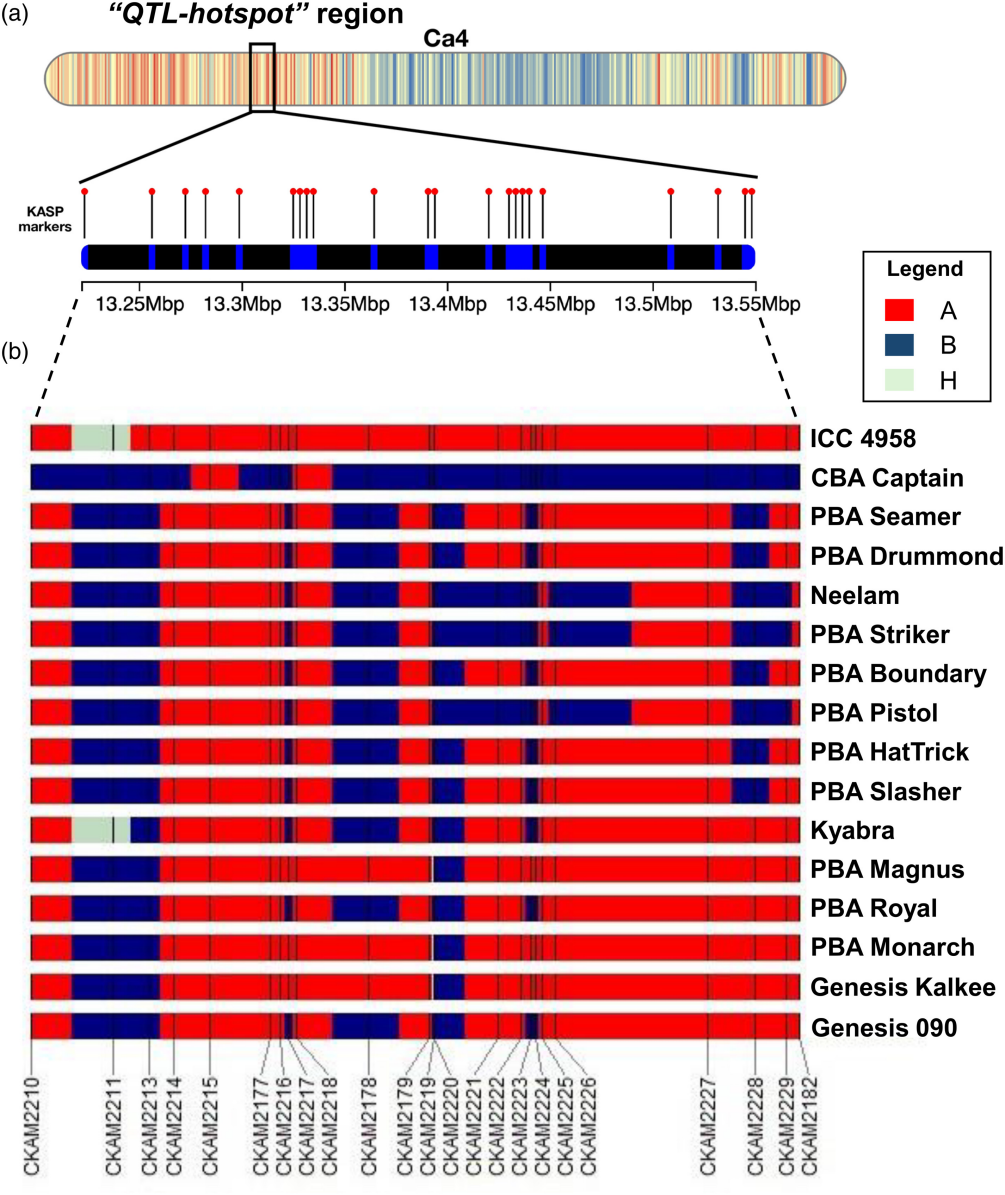
Validation of superior alleles within the ‘*QTL‐hotspot*’ region using KASP markers. (a) Schematic representation of the ‘*QTL‐hotspot*’ region on Ca4 of chickpea genome. The highlighted black box zooms in on the distribution of SNPs converted to Kompetitive allele‐specific PCR (KASP) assay markers. (b) Genotypic variations of ICC 4958 and 15 Australian chickpea cultivars based on 23 KASP markers within the ‘*QTL‐hotspot*’ region. Different colours indicate distinct alleles (A, B, and heterozygous H), providing a comparative overview of allelic diversity across the cultivars.

The Kompetitive Allele‐Specific PCR (KASP) assay has become a widely used technology for developing trait‐specific markers due to its cost‐effectiveness, high genotyping accuracy, reliability, and reproducibility (Dipta *et al*., 2024). This study used KASP markers linked to 23 SNPs within the ~300 kb ‘*QTL‐hotspot*’ region to assess the presence or absence of superior haplotypes across 15 Australian chickpea cultivars (Table S18). Of the KASP markers tested, 20 showed polymorphism between ICC 4958 and the Australian cultivars. Notably, four KASP markers—CKAM2177, CKAM2217, CKAM2221, and CKAM2226—targeting SNPs in key genes *CaARD1* (encoding 1,2‐dihydroxy‐3‐keto‐5‐methylthiopentene dioxygenase), *CaTIFY4b*, *CaLRX2*, and *CaKIP1*, respectively, effectively differentiated the Australian cultivars based on the presence or absence of superior haplotypes (Figure 6a,b). For CKAM2217, the allele ‘C/C’, associated with the superior haplotype *CaTIFY4b‐H2*, was absent in all Australian desi cultivars, Genesis 090 and PBA Royal. Similarly, CBA Captain, PBA Striker, PBA Pistol, and Neelam lacked the allele ‘C/C’ linked to the superior haplotype *CaLRX2‐H4* and instead carried the allele ‘G/G’. For CKAM2226, the allele ‘A/A’ associated with the superior haplotype *CaKIP1‐H7* was also absent in CBA Captain, PBA Striker, PBA Pistol, and Neelam. Furthermore, CBA Captain, a leading Australian chickpea cultivar, carried the allele ‘T/T’ and lacked the allele ‘C/C’ for CKAM2177, which is associated with the superior haplotype *CaARD1‐H5* for *Ca_04557* (*CaARD1*) gene. The absence of superior haplotypes for key genes within the ‘*QTL‐hotspot*’ region across several leading Australian chickpea cultivars, as validated by KASP markers, highlights an opportunity for haplotype‐based breeding. Introgression of these beneficial haplotypes from ICC 4958 could significantly enhance the drought adaptation of Australian chickpeas.

## Discussion

Since the release of Australia's first commercial chickpea variety in 1978 (Bullard *et al*., 2005), the industry has faced numerous challenges, including changes in agricultural practices, disease pressures, abiotic stresses, shifting export market demands, and soil constraints. Understanding the genetic makeup of Australian chickpeas is critical for identifying key genetic factors that regulate yield, stress tolerance, and disease resistance. Additionally, broadening genetic diversity by incorporating beneficial alleles from diverse backgrounds provides a solid foundation for future breeding efforts. Such strategies will help develop resilient and high‐yielding Australian chickpea varieties suited to evolving agricultural needs. This study presents high‐quality genome assemblies and features of 15 commercially grown Australian chickpea cultivars, confirming the absence of superior haplotypes for key genes within the ‘*QTL‐hotspot*’ region and highlighting opportunities to introduce novel genetic diversity to enhance drought adaptation.

A high‐quality reference genome is essential for understanding genome organization and accurately identifying regions linked to important agronomic traits (Garg *et al*., 2024). In this study, we present high‐quality genome assemblies of 15 Australian chickpea cultivars generated using stLFR technology, offering a robust foundation for dissecting the genetic architecture of key agronomic traits and advancing chickpea breeding in Australia. The stLFR technology, which employs DNA co‐barcoding to assign identical barcode sequences to sub‐fragments of the original long DNA molecule, provides a cost‐effective alternative to long‐read sequencing by leveraging second‐generation sequencing with a low error rate (Wang *et al*., 2023a). The reference‐guided genome assemblies of 15 cultivars were developed with an average size of 468 Mb, with ~93% of sequences anchored to chromosomes. The assembled genomes contained ~99% complete BUSCOs, indicating their high quality. Additionally, the integration of homology searches, *ab initio* predictions, and transcript evidence enabled the identification of 31 056 genes, exceeding previously reported gene counts (Garg *et al*., 2022; Varshney *et al*., 2013).

Pan‐genomes provide a more comprehensive representation of genetic diversity than single reference genomes and are widely used to capture genomic variations linked to important traits. In recent years, pan‐genome studies have been conducted for various crops (Jiao *et al*., 2025; Liu *et al*., 2020, Liu *et al*., 2022a; Shang *et al*., 2022; Yang *et al*., 2022). In this study, we constructed a pan‐genome using the 15 newly assembled Australian chickpea genomes and the ICC 4958 and CDC Frontier reference genomes. We identified 34 345 gene families, of which 13 986 were dispensable gene families that were enriched for stress adaptation‐related functions, including defence response, DNA repair, telomere maintenance, and cell wall modification, consistent with previous studies (Chen *et al*., 2023; Khan *et al*., 2024). These findings highlight the role of dispensable genes in conferring adaptive advantages to varying environmental conditions and biotic stresses.

Analysis of genetic diversity across 15 Australian chickpea cultivars identified 2 473 162 non‐redundant SNPs and 213 633 InDels distributed across eight chromosomes. Notably, CBA Captain exhibited the highest number of SNPs (1 538 962) and InDels (122 599), which may contribute to its superior agronomic performance in Australia. This extensive genetic variation likely underpins key traits, such as optimal plant height, pod positioning, and lodging resistance, essential for successful cultivation and harvestability (Hobson *et al*., 2021). The SV analysis across the 15 Australian chickpea cultivars and ICC 4958 identified large‐scale insertions, deletions, translocations, inversions, and duplications. For instance, a 95.62 kb duplication on chromosome 7 in PBA Slasher contained a gene encoding E3 ubiquitin–protein ligase MBR2‐like protein, a key regulator of cellular processes that tags proteins for degradation via the ubiquitin pathway, influencing organ size, flowering time, and stress responses (Iñigo *et al*., 2012; Kelley, 2018). Furthermore, a 118.86 kb deletion in Genesis 090 resulted in the loss of six genes, including those encoding E3 ubiquitin–protein ligase RLIM‐like proteins and the anaphase‐promoting complex subunit 11 RING‐H2 finger protein, both critical for ubiquitination and regulation of plant developmental processes (Heyman and De Veylder, 2012; Shu and Yang, 2017). To leverage this genetic diversity within the pan‐genome, a graph‐based genome reference was constructed by integrating SVs from the 15 Australian cultivars and ICC 4958 into the linear reference sequence (Cicar.CDCFrontier_v2.0). This graph genome mitigates reference bias and enables more comprehensive representation of SV, providing a robust platform for SV‐based association studies and facilitating the identification of genetic variations underlying important agronomic traits.

Despite the observed genetic diversity, the breeding history of Australian chickpea cultivars remains relatively narrow, largely due to the repeated use of a limited number of parental lines in breeding programmes. For example, among the desi cultivars analysed in this study, ICC 3996 was a common parent contributing to the development of PBA Striker, PBA Slasher, and PBA Boundary. Likewise, most kabuli cultivars in this study were derived primarily from International Centre for Agricultural Research in the Dry Areas (ICARDA) nurseries, reflecting limited genetic variability (Thudi *et al*., 2016; Varshney *et al*., 2021). For example, Genesis 090 is a direct introduction of FLIP94‐090C and Genesis Kalkee is a reselection of the ICARDA line FLIP97‐114C. This narrow genetic diversity poses significant challenges to the adaptability and resilience of Australian chickpeas to various abiotic stresses (e.g. drought, heat, and cold) and biotic stresses (e.g. Ascochyta blight, *Phytophthora* root rot, and *Fusarium* wilt). Addressing these challenges requires introgressing novel alleles for yield, stress tolerance, and disease resistance from genetically diverse accessions. One such source is ICC 4958, a well‐characterized drought‐tolerant cultivar (Kashiwagi *et al*., 2006), which carries the ‘*QTL‐hotspot*’ region associated with key drought‐adaptive traits (Barmukh *et al*., 2022b). In this study, haplotype analysis of key genes underlying the ‘*QTL‐hotspot*’ region revealed that most Australian chickpea cultivars lack superior haplotypes such as *CaARD1‐H7*, *CaTIFY4b‐H2*, *CaTSJT1‐H2*, and *CaLRX2‐H4*, among others. This absence was further validated using KASP markers targeting SNPs in genes, such as *CaARD1*, *CaTIFY4b*, *CaLRX2*, and *CaKIP1*. These findings highlight a major opportunity to introgress beneficial alleles from ICC 4958 into Australian chickpea cultivars to enhance drought adaptation.

Broadening the genetic base of Australian chickpeas by introducing beneficial alleles from diverse germplasm accessions will enhance climate resilience, ensuring high productivity under increasingly variable environmental conditions. This approach aligns with global efforts to improve food and nutritional security, ultimately supporting the long‐term sustainability of chickpea production in Australia.

## Materials and methods

### Plant material

This study evaluated 15 commercially grown Australian chickpea cultivars. Among the desi types, CBA Captain (released in 2020), PBA Drummond (2018), and PBA Seamer (2016) are high‐yielding cultivars with broad adaptation and favourable agronomic traits such as erect or semi‐erect growth habits, mid‐flowering, and early to mid‐maturity. Neelam (2013), PBA Striker (2012), PBA Boundary (2011), PBA Pistol (2011), PBA HatTrick (2009), and PBA Slasher (2009) also offer good adaptation across different Australian regions and are known for desirable traits, such as plant architecture, flowering time, maturity, and milling quality. Kyabra (2005) is another widely grown desi cultivar known for its high yield and seed quality.

The kabuli cultivars include PBA Magnus (2020) and PBA Royal (2019), which are high‐yielding varieties suited to medium to high rainfall environments. PBA Monarch (2013), Genesis Kalkee (2012), and Genesis 090 (2005; introduced from ICARDA) are also well‐adapted kabuli types, exhibiting a range of seed sizes, flowering times, and plant types suited for Australian production regions.

### Genome sequencing and assembly

Seeds of the 15 chickpea cultivars were obtained from the Australian Grains GeneBank (AGG) and sown in pots under near‐optimal glasshouse conditions. Genomic DNA was extracted from leaves of 15‐day‐old seedlings using the Qiagen DNeasy Plant Mini Kit following the manufacturer's protocols. DNA quality was assessed on a 0.8% agarose gel while DNA quantity was measured using a Qubit® 2.0 fluorometer (Life Technologies, ThermoFisher Scientific, Carlsbad, CA, USA). High‐molecular‐weight DNA was used for library preparation and sequencing. According to the manufacturer's instructions, stLFR sequencing libraries were prepared using the MGIEasy stLFR Library Prep Kit (MGI, Shenzhen, China) and sequenced using the DNBSeq‐T7 platform. The stLFR sequencing data were processed using the stlfr2supernova pipeline (https://github.com/BGI‐Qingdao/stlfr2supernova_pipeline; Wang *et al*., 2019) and assembled with the Supernova assembler v2.1.1 (https://github.com/10XGenomics/supernova; Weisenfeld *et al*., 2017) using default parameters. Each of the 15 genomes was assembled independently. The resulting preliminary assemblies were further scaffolded using RagTag v1.1.0 (https://github.com/malonge/RagTag; Alonge *et al*., 2022) with CDC Frontier as the reference genome (Cicar.CDCFrontier_v2.0; Garg *et al*., 2022). The completeness of the reference‐guided assemblies was assessed using BUSCO (v5.2.2; Manni *et al*., 2021) with the ‘embryophyta_odb10’ dataset. The ‘chromosome anchoring rate’ was calculated as the percentage of assembled sequences that could be aligned and anchored to the chromosomes of the reference genome.

### Identification of repeats

Repeat elements in the genome assemblies were identified and annotated using a combination of *de novo* and homology‐based approaches. Two repeat detection programmes were used: Extensive *de novo* TE Annotator (EDTA) v2.0.1 (Ou *et al*., 2019) and TransposonPSI (http://transposonpsi.sourceforge.net/). EDTA integrates multiple tools, including LTRharvest, LTR_FINDER, LTR_retriever, TIR‐Learner, HelitronScanner, RepeatModeler, and RepeatMasker, alongside custom filtering scripts for *de novo* identification of different transposable element (TE) classes. The results were compiled into a comprehensive TE library for each genotype. Repeat elements in the genomes were annotated using RepeatMasker (version 4.0.5) (Tarailo‐Graovac and Chen, 2009) with the custom TE libraries.

### Gene prediction and annotation

Gene models were predicted for each genome using an integrative approach combining *ab initio* gene prediction, homology‐based prediction, and transcript‐based evidence. For *ab initio* gene prediction, BRAKER v2.1.6 (Brůna *et al*., 2021) was used, incorporating Augustus v3.3.3 (Stanke *et al*., 2008) and GeneMark‐ES/ET/EP+ (Brůna *et al*., 2020) to train gene models using protein sequences from Phytozome and UniProt, and publicly available *C. arietinum* RNA‐seq data. For homology‐based prediction, protein sequences from Phytozome v13 (*Glycine max*, *Arabidopsis thaliana*, *Cajanus cajan*, and *Medicago truncatula*) and UniProtKB/Swiss‐Prot Viridiplantae were aligned to each genome assembly using BLAT v.36 and refined with GeneWise v.2.4.1 (Birney *et al*., 2004). Transcriptome‐based evidence was derived from publicly available RNA‐seq datasets for different chickpea cultivars, including multiple varieties used in this study (Table S19). These datasets were aligned to each genome using HISAT2 v2.1.0 (Kim *et al*., 2019), and transcript assemblies were generated with StringTie v2.0 (Pertea *et al*., 2015). These predictions were integrated through two iterations of the MAKER2 v2.31.11 pipeline (Holt and Yandell, 2011), combining *ab initio*, homology‐based, and evidence‐based gene models. The final MAKER output was filtered based on Annotation Edit Distance (AED), retaining only gene models with AED values <0.5. The following criteria were applied to remove transposon‐related genes: (i) genes overlapping repetitive regions (>60%) and transposase domain regions (>60%), (ii) genes overlapping repetitive regions (>60%) and annotated as transposon‐associated, and (iii) genes identified as transposon‐associated at least two independent databases. Predicted gene models were assigned putative functions using BLASTP (E‐value <1E‐05) against the Swiss‐Prot, TrEMBL, and NCBI non‐redundant protein databases. Conserved domains and motifs were identified using InterProScan v5.39–77.0 (Jones *et al*., 2014), and Gene Ontology (GO) IDs were assigned accordingly. Pathway annotation was performed using BLAST searches against the Kyoto Encyclopedia of Genes and Genomes (KEGG) database. For resistance gene identification, the RGAugury pipeline (version 2017–10‐21; Li *et al*., 2016) was employed, classifying genes into subfamilies based on the presence or absence of specific domains.

Additionally, ribosomal RNA (rRNA) genes were identified using BLASTN (*E*‐value <1E‐05) with *Arabidopsis thaliana* and *Oryza sativa* rRNA sequences as queries. MicroRNAs (miRNAs) and small nucleolar RNAs (snoRNA) were detected using Infernal v1.1.3 (Nawrocki and Eddy, 2013). Transfer RNAs (tRNA) were predicted using tRNAscan‐SE v2.0 (Chan *et al*., 2021).

### Core and dispensable genes

Orthologous gene clusters were identified in the assembled genomes of 17 chickpea cultivars (15 Australian cultivars, ICC 4958, and CDC Frontier) using Orthofinder v2.5.4 (Emms and Kelly, 2019) with default parameters. Gene models for CDC Frontier and ICC 4958 were re‐predicted using the same workflow described earlier to ensure consistency and avoid methodological bias. These gene models were used exclusively for core and dispensable genome estimations. Gene families were classified as ‘core’ if present in all cultivars, ‘soft‐core’ if present in all but one cultivar or ‘variable/dispensable’ if absent in at least one cultivar. Pan‐genome and core genome sizes were analysed and modelled using the nlsLM function in R, as described by Khan *et al*. (2024). In the modelling curve, the plateau point was defined as the stage at which adding new genomes resulted in an increase of <0.5% in the total number of gene families.

### Identification of variations

Short variations, including SNPs and InDels (<50 bp), were identified by aligning the stLFR sequencing data for all the Australian cultivars to the CDC Frontier v2.0 reference genome using BWA v0.7.17 (Li and Durbin, 2009). Uniquely aligned reads were extracted using samtools and used for variant calling with bcftools v1.19 (Danecek *et al*., 2021). Variants were filtered based on the following criteria: variant quality ≥20 and minimum read depth of 50. Large SVs (≥50 bp), including large deletions, insertions, inversions, duplications, and translocations, were identified via pairwise genome alignments using minimap2 v2.26 (Li, 2021). Aligned data were processed in haploid mode using SVIM‐asm v1.0.3 (Heller and Vingron, 2021) to detect SVs. These variations were merged across cultivars using SURVIVOR v1.0.7 (Jeffares *et al*., 2017) with a minimum SV length of 50 bp, taking strand information into account. A graph‐based pan‐genome was constructed using the ‘construct' subcommand of VG v1.37.0 (Garrison *et al*., 2018) with default parameters by integrating SVs from 15 Australian genome assemblies with CDC Frontier as the backbone. Whole‐genome synteny plots were generated using GENESPACE v1.1.10 (Lovell *et al*., 2022).

### Haplotype analysis

Haplotype analysis was conducted for 26 genes within the ‘*QTL‐hotspot*’ region using the SNP dataset for 3171 chickpea cultivated accessions generated in a previous study (Varshney *et al*., 2021). Haplotypes containing heterozygous SNPs were excluded from the analysis. Phenotypic data for 100‐seed weight, recorded at ICRISAT during the 2015–16 cropping season, were utilized for haplotype‐phenotype association analysis (Varshney *et al*., 2021). The mean 100‐seed weight values for each haplotype group were compared to identify superior haplotypes. Statistical significance among haplotype groups was determined using Duncan's multiple range test in GenStat (https://vsni.co.uk/software/genstat/), with different letters in the graphs indicating significant differences at *P* < 0.05.

### Validation using KASP assay markers

About 23 KASP assay markers targeting 23 SNPs within the ~300‐kb ‘*QTL‐hotspot*’ region were used for validation (Table S18). The selected markers CKAM2210, CKAM2211, CKAM2213, CKAM2214, CKAM2215, CKAM2177, CKAM2216, CKAM2217, CKAM2218, CKAM2178, CKAM2179, CKAM2219, CKAM2220, CKAM2221, CKAM2222, CKAM2223, CKAM2224, CKAM2225, CKAM2226, CKAM2227, CKAM2228, CKAM2229, and CKAM2182 were used to assess the presence or absence of superior alleles in 15 Australian chickpea cultivars. Among these, CKAM2210 and CKAM2182 were used as flanking markers for the ‘*QTL‐hotspot*’ region.

## Conflict of interest

The authors declare that they have no competing interests.

## Author contributions

R.K.V. conceived and designed the experiments. V.G., R.B., Y.H., B.Y., S.B., and X.L. analysed the data. A.C., K.H., Y.J., S.K., M.A.A., M.H., S.N., D.L.S., K.H.M.S., and C.L. contributed to plant materials, reagents, and sequencing. V.G., R.B., K.H.M.S., and R.K.V. wrote the manuscript. All authors read and approved the manuscript.

## Supporting information


**Figure S1** GC content distribution across different Australian chickpea cultivars.
**Figure S2** Length distribution of various gene features across different chickpea cultivars.
**Figure S3** Gene Ontology (GO) enrichment analysis of core and dispensable gene families.
**Figure S4** Genome‐wide distribution of InDels across the Australian chickpea cultivars.

**Table S1** Details of the 15 Australian chickpea cultivars used in this study.
**Table S2** Summary of raw sequencing data generated.
**Table S3** Summary of non‐coding RNAs identified in the Australian chickpea cultivars.
**Table S4** Summary of ortholog analysis across 17 chickpea cultivars.
**Table S5** Statistics of SNPs identified in the Australian chickpea cultivars.
**Table S6** Statistics of InDels identified in the Australian chickpea cultivars.
**Table S7** Functional annotation of SNPs and InDels identified in the Australian chickpea cultivars.
**Table S8** Summary of structural variations identified in the Australian chickpea cultivars.
**Table S9** Genome‐wide SNPs and small InDels identified in flowering time‐related genes across ICC 4958 and 15 Australian chickpea cultivars.
**Table S10** Genome‐wide SNPs and small InDels identified in resistance genes across ICC 4958 and 15 Australian chickpea cultivars.
**Table S11** Genome‐wide SNPs and small InDels identified in seed size‐related genes across ICC 4958 and 15 Australian chickpea cultivars.
**Table S12** SNPs and InDels identified within the ‘*QTL‐hotspot*’ region across ICC 4958 and 15 Australian chickpea cultivars.
**Table S13** Haplotypes of 22 genes located within the ‘*QTL‐hotspot*’ region in ICC 4958 and 15 Australian chickpea cultivars.
**Table S14** Haplotype distribution and frequency of the *CaTIFY4b* gene across a composite chickpea collection.
**Table S15** Haplotype distribution and frequency of the *CaTSJT1* gene across a composite chickpea collection.
**Table S16** Haplotype distribution and frequency of the *CaLRX2* gene across a composite chickpea collection.
**Table S17** Haplotype distribution and frequency of the *CaKIP1* gene across a composite chickpea collection.
**Table S18** List of primers used for the development of KASP assays.
**Table S19** List of publicly available RNA‐seq datasets used for gene prediction.

## Data Availability

The raw sequencing data generated in the study have been deposited in NCBI with the BioProject ID ‘PRJNA1225167’. The genome assemblies and annotations are available on Figshare at https://doi.org/10.6084/m9.figshare.28632494.v1.

## References

[pbi70192-bib-0001] Alonge, M. , Lebeigle, L. , Kirsche, M. , Jenike, K. , Ou, S. , Aganezov, S. , Wang, X. *et al*. (2022) Automated assembly scaffolding using RagTag elevates a new tomato system for high‐throughput genome editing. Genome Biol. 23, 258. 36522651 10.1186/s13059-022-02823-7 PMC9753292

[pbi70192-bib-0002] Alonge, M. , Wang, X. , Benoit, M. , Soyk, S. , Pereira, L. , Zhang, L. , Suresh, H. *et al*. (2020) Major impacts of widespread structural variation on gene expression and crop improvement in tomato. Cell 182, 145–161. 32553272 10.1016/j.cell.2020.05.021 PMC7354227

[pbi70192-bib-0003] Bai, Y. , Meng, Y. , Huang, D. , Qi, Y. and Chen, M. (2011) Origin and evolutionary analysis of the plant‐specific TIFY transcription factor family. Genomics 98, 128–136. 21616136 10.1016/j.ygeno.2011.05.002

[pbi70192-bib-0004] Barmukh, R. , Roorkiwal, M. , Dixit, G.P. , Bajaj, P. , Kholova, J. , Smith, M.R. , Chitikineni, A. *et al*. (2022a) Characterization of “QTL‐hotspot” introgression lines reveals physiological mechanisms and candidate genes associated with drought adaptation in chickpea. J. Exp. Bot. 73, 7255–7272. 36006832 10.1093/jxb/erac348 PMC9730794

[pbi70192-bib-0005] Barmukh, R. , Roorkiwal, M. , Garg, V. , Khan, A.W. , German, L. , Jaganathan, D. , Chitikineni, A. *et al*. (2022b) Genetic variation in CaTIFY4b contributes to drought adaptation in chickpea. Plant Biotechnol. J. 20, 1701–1715. 35534989 10.1111/pbi.13840 PMC9398337

[pbi70192-bib-0006] Berger, J.D. , Turner, N.C. , Siddique, K.H.M. , Knights, E.J. , Brinsmead, R.B. , Mock, I. , Edmondson, C. *et al*. (2004) Genotype by environment studies across Australia reveal the importance of phenology for chickpea (*Cicer arietinum* L.) improvement. Aust. J. Agric. Res. 55, 1071.

[pbi70192-bib-0007] Bharadwaj, C. , Tripathi, S. , Soren, K.R. , Thudi, M. , Singh, R.K. , Sheoran, S. , Roorkiwal, M. *et al*. (2021) Introgression of “QTL‐hotspot” region enhances drought tolerance and grain yield in three elite chickpea cultivars. Plant Genome 14, e20076. 33480153 10.1002/tpg2.20076 PMC12807433

[pbi70192-bib-0008] Birney, E. , Clamp, M. and Durbin, R. (2004) GeneWise and Genomewise. Genome Res. 14, 988–995. 15123596 10.1101/gr.1865504 PMC479130

[pbi70192-bib-0009] Bose, U. , Buck, S. , Sirault, X. , Bahmani, M. , Byrne, K. , Stockwell, S. , McWilliam, S. *et al*. (2024) Chickpea proteome analysis reveals genotype‐dependent variations associated with seed traits. J. Agric. Food Chem. 72, 27030–27042. 39570711 10.1021/acs.jafc.4c07669 PMC11622230

[pbi70192-bib-0010] Brůna, T. , Hoff, K.J. , Lomsadze, A. , Stanke, M. and Borodovsky, M. (2021) BRAKER2: automatic eukaryotic genome annotation with GeneMark‐EP+ and AUGUSTUS supported by a protein database. NAR Genomics Bioinform. 3, lqaa108. 10.1093/nargab/lqaa108 PMC7787252 33575650

[pbi70192-bib-0011] Brůna, T. , Lomsadze, A. and Borodovsky, M. (2020) GeneMark‐EP+: eukaryotic gene prediction with self‐training in the space of genes and proteins. NAR Genomics and Bioinformatics 2, lqaa026. 32440658 10.1093/nargab/lqaa026 PMC7222226

[pbi70192-bib-0012] Bullard, G.K. , Roughley, R.J. and Pulsford, D.J. (2005) The legume inoculant industry and inoculant quality control in Australia: 1953–2003. Aust. J. Exp. Agric. 45, 127.

[pbi70192-bib-0013] Chai, G. , Liu, H. , Zhang, Y. , Wang, C. , Xu, H. , He, G. , Meng, J. *et al*. (2024) Integration of C3H15‐mediated transcriptional and post‐transcriptional regulation confers thermotolerance in Arabidopsis. Plant J. 119, 1558–1569. 38865085 10.1111/tpj.16877

[pbi70192-bib-0014] Chan, P.P. , Lin, B.Y. , Mak, A.J. and Lowe, T.M. (2021) tRNAscan‐SE 2.0: improved detection and functional classification of transfer RNA genes. Nucleic Acids Res. 49, 9077–9096. 34417604 10.1093/nar/gkab688 PMC8450103

[pbi70192-bib-0015] Chapman, M.A. , He, Y. and Zhou, M. (2022) Beyond a reference genome: pangenomes and population genomics of underutilized and orphan crops for future food and nutrition security. New Phytol. 234, 1583–1597. 35318683 10.1111/nph.18021 PMC9994440

[pbi70192-bib-0016] Chattopadhyay, D. and Francis, A. (2021) A draft genome assembly of Cicer arietinum accession ICC4958_v3.0 [Dataset]. figshare. 10.6084/M9.FIGSHARE.14579274.V1

[pbi70192-bib-0017] Chen, J. , Liu, Y. , Liu, M. , Guo, W. , Wang, Y. , He, Q. , Chen, W. *et al*. (2023) Pangenome analysis reveals genomic variations associated with domestication traits in broomcorn millet. Nat. Genet. 55, 2243–2254. 38036791 10.1038/s41588-023-01571-z PMC10703678

[pbi70192-bib-0018] Choudhary, A.K. , Jain, S.K. , Dubey, A.K. , Kumar, J. , Sharma, M. , Gupta, K.C. , Sharma, L.D. *et al*. (2023) Conventional and molecular breeding for disease resistance in chickpea: status and strategies. Biotechnol. Genet. Eng. Rev. 39, 193–224. 35959728 10.1080/02648725.2022.2110641

[pbi70192-bib-0019] Danecek, P. , Bonfield, J.K. , Liddle, J. , Marshall, J. , Ohan, V. , Pollard, M.O. , Whitwham, A. *et al*. (2021) Twelve years of SAMtools and BCFtools. GigaScience 10, giab008. 33590861 10.1093/gigascience/giab008 PMC7931819

[pbi70192-bib-0020] Dey, A. , Jangir, N. , Verma, D. , Shekhawat, R.S. , Yadav, P. and Sadhukhan, A. (2025) Foliar application of nano Urea enhances vegetative growth of Arabidopsis thaliana over equimolar bulk urea through higher induction of biosynthesis genes but suppression of nitrogen uptake and senescence genes. Plant Growth Regul. 105, 833–859.

[pbi70192-bib-0021] Ding, L. , Xu, H. , Yi, H. , Yang, L. , Kong, Z. , Zhang, L. , Xue, S. *et al*. (2011) Resistance to hemi‐biotrophic *F. graminearum* infection is associated with coordinated and ordered expression of diverse defense signaling pathways. PLoS One 6, e19008. 21533105 10.1371/journal.pone.0019008 PMC3080397

[pbi70192-bib-0022] Dipta, B. , Sood, S. , Mangal, V. , Bhardwaj, V. , Thakur, A.K. , Kumar, V. and Singh, B. (2024) KASP: a high‐throughput genotyping system and its applications in major crop plants for biotic and abiotic stress tolerance. Mol. Biol. Rep. 51, 508. 38622474 10.1007/s11033-024-09455-z

[pbi70192-bib-0023] Emms, D.M. and Kelly, S. (2019) OrthoFinder: phylogenetic orthology inference for comparative genomics. Genome Biol. 20, 238. 31727128 10.1186/s13059-019-1832-y PMC6857279

[pbi70192-bib-0024] FAOSTAT . (2023) FAOSTAT database. Retrieved January 25, 2025, from http://www.fao.org/faostat/en/#data/QCL

[pbi70192-bib-0025] Garg, V. , Bohra, A. , Mascher, M. , Spannagl, M. , Xu, X. , Bevan, M.W. , Bennetzen, J.L. *et al*. (2024) Unlocking plant genetics with telomere‐to‐telomere genome assemblies. Nat. Genet. 56, 1788–1799. 39048791 10.1038/s41588-024-01830-7

[pbi70192-bib-0026] Garg, V. , Dudchenko, O. , Wang, J. , Khan, A.W. , Gupta, S. , Kaur, P. , Han, K. *et al*. (2022) Chromosome‐length genome assemblies of six legume species provide insights into genome organization, evolution, and agronomic traits for crop improvement. J. Adv. Res. 42, 315–329. 36513421 10.1016/j.jare.2021.10.009 PMC9788938

[pbi70192-bib-0027] Garrison, E. , Sirén, J. , Novak, A.M. , Hickey, G. , Eizenga, J.M. , Dawson, E.T. , Jones, W. *et al*. (2018) Variation graph toolkit improves read mapping by representing genetic variation in the reference. Nat. Biotechnol. 36, 875–879. 30125266 10.1038/nbt.4227 PMC6126949

[pbi70192-bib-0028] Ge, L. , Yu, J. , Wang, H. , Luth, D. , Bai, G. , Wang, K. and Chen, R. (2016) Increasing seed size and quality by manipulating BIG SEEDS1 in legume species. Proc. Natl. Acad. Sci. USA 113, 12414–12419. 27791139 10.1073/pnas.1611763113 PMC5098654

[pbi70192-bib-0029] Golicz, A.A. , Batley, J. and Edwards, D. (2016) Towards plant pangenomics. Plant Biotechnol. J. 14, 1099–1105. 26593040 10.1111/pbi.12499 PMC11388911

[pbi70192-bib-0030] Hanzawa, Y. , Takahashi, T. and Komeda, Y. (1997) ACL5: an Arabidopsis gene required for internodal elongation after flowering. Plant J. 12, 863–874. 9375398 10.1046/j.1365-313x.1997.12040863.x

[pbi70192-bib-0031] He, Q. , Tang, S. , Zhi, H. , Chen, J. , Zhang, J. , Liang, H. , Alam, O. *et al*. (2023) A graph‐based genome and pan‐genome variation of the model plant Setaria. Nat. Genet. 55, 1232–1242. 37291196 10.1038/s41588-023-01423-w PMC10335933

[pbi70192-bib-0032] Heller, D. and Vingron, M. (2021) SVIM‐asm: structural variant detection from haploid and diploid genome assemblies. Bioinformatics 36, 5519–5521. 33346817 10.1093/bioinformatics/btaa1034 PMC8016491

[pbi70192-bib-0033] Heyman, J. and De Veylder, L. (2012) The anaphase‐promoting complex/cyclosome in control of plant development. Mol. Plant 5, 1182–1194. 23034505 10.1093/mp/sss094

[pbi70192-bib-0034] Hobson, K. , Dron, N. , Ryan, M. , Martin, W. , Graham, N. , Warren, A. , Davidson, J. *et al*. (2021) CBA Captain: a new desi variety for the northern region – Grains Research and Development Corporation. https://grdc.com.au/resources‐and‐publications/grdc‐update‐papers/tab‐content/grdc‐update‐papers/2021/02/cba‐captain‐a‐new‐desi‐variety‐for‐the‐northern‐region

[pbi70192-bib-0035] Holt, C. and Yandell, M. (2011) MAKER2: an annotation pipeline and genome‐database management tool for second‐generation genome projects. BMC Bioinformatics 12, 491. 22192575 10.1186/1471-2105-12-491 PMC3280279

[pbi70192-bib-0036] Horiguchi, G. , Kim, G.‐T. and Tsukaya, H. (2005) The transcription factor AtGRF5 and the transcription coactivator AN3 regulate cell proliferation in leaf primordia of *Arabidopsis thaliana* . Plant J. 43, 68–78. 15960617 10.1111/j.1365-313X.2005.02429.x

[pbi70192-bib-0037] Hufnagel, B. , Marques, A. , Soriano, A. , Marquès, L. , Divol, F. , Doumas, P. , Sallet, E. *et al*. (2020) High‐quality genome sequence of white lupin provides insight into soil exploration and seed quality. Nat. Commun. 11, 492. 31980615 10.1038/s41467-019-14197-9 PMC6981116

[pbi70192-bib-0038] Iñigo, S. , Giraldez, A.N. , Chory, J. and Cerdán, P.D. (2012) Proteasome‐mediated turnover of Arabidopsis MED25 is coupled to the activation of FLOWERING LOCUS T transcription. Plant Physiol. 160, 1662–1673. 22992513 10.1104/pp.112.205500 PMC3490578

[pbi70192-bib-0039] Jayakodi, M. , Lu, Q. , Pidon, H. , Rabanus‐Wallace, M.T. , Bayer, M. , Lux, T. , Guo, Y. *et al*. (2024) Structural variation in the pangenome of wild and domesticated barley. Nature 636, 654–662. 39537924 10.1038/s41586-024-08187-1 PMC11655362

[pbi70192-bib-0040] Jeffares, D.C. , Jolly, C. , Hoti, M. , Speed, D. , Shaw, L. , Rallis, C. , Balloux, F. *et al*. (2017) Transient structural variations have strong effects on quantitative traits and reproductive isolation in fission yeast. Nat. Commun. 8, 14061. 28117401 10.1038/ncomms14061 PMC5286201

[pbi70192-bib-0041] Jiao, C. , Xie, X. , Hao, C. , Chen, L. , Xie, Y. , Garg, V. , Zhao, L. *et al*. (2025) Pan‐genome bridges wheat structural variations with habitat and breeding. Nature 637, 384–393. 39604736 10.1038/s41586-024-08277-0

[pbi70192-bib-0042] Jones, P. , Binns, D. , Chang, H.‐Y. , Fraser, M. , Li, W. , McAnulla, C. , McWilliam, H. *et al*. (2014) InterProScan 5: genome‐scale protein function classification. Bioinformatics 30, 1236–1240. 24451626 10.1093/bioinformatics/btu031 PMC3998142

[pbi70192-bib-0043] Kale, S.M. , Jaganathan, D. , Ruperao, P. , Chen, C. , Punna, R. , Kudapa, H. , Thudi, M. *et al*. (2015) Prioritization of candidate genes in “QTL‐hotspot” region for drought tolerance in chickpea (*Cicer arietinum* L.). Sci. Rep. 5, 15296. 26478518 10.1038/srep15296 PMC4609953

[pbi70192-bib-0044] Kashiwagi, J. , Krishnamurthy, L. , Crouch, J.H. and Serraj, R. (2006) Variability of root length density and its contributions to seed yield in chickpea (*Cicer arietinum* L.) under terminal drought stress. Field Crop Res. 95, 171–181.

[pbi70192-bib-0045] Kelley, D.R. (2018) E3 ubiquitin ligases: Key regulators of hormone signaling in plants. Mol. Cell. Proteomics 17, 1047–1054. 29514858 10.1074/mcp.MR117.000476 PMC5986243

[pbi70192-bib-0046] Khan, A.W. , Garg, V. , Roorkiwal, M. , Golicz, A.A. , Edwards, D. and Varshney, R.K. (2020) Super‐pangenome by integrating the wild side of a species for accelerated crop improvement. Trends Plant Sci. 25, 148–158. 31787539 10.1016/j.tplants.2019.10.012 PMC6988109

[pbi70192-bib-0047] Khan, A.W. , Garg, V. , Sun, S. , Gupta, S. , Dudchenko, O. , Roorkiwal, M. , Chitikineni, A. *et al*. (2024) Cicer super‐pangenome provides insights into species evolution and agronomic trait loci for crop improvement in chickpea. Nat. Genet. 56, 1225–1234. 38783120 10.1038/s41588-024-01760-4

[pbi70192-bib-0048] Kim, D. , Paggi, J.M. , Park, C. , Bennett, C. and Salzberg, S.L. (2019) Graph‐based genome alignment and genotyping with HISAT2 and HISAT‐genotype. Nat. Biotechnol. 37, 907–915. 31375807 10.1038/s41587-019-0201-4 PMC7605509

[pbi70192-bib-0049] Li, H. (2021) New strategies to improve minimap2 alignment accuracy. Bioinformatics 37, 4572–4574. 34623391 10.1093/bioinformatics/btab705 PMC8652018

[pbi70192-bib-0050] Li, H. and Durbin, R. (2009) Fast and accurate short read alignment with Burrows‐Wheeler transform. Bioinformatics 25, 1754–1760. 19451168 10.1093/bioinformatics/btp324 PMC2705234

[pbi70192-bib-0051] Li, P. , Quan, X. , Jia, G. , Xiao, J. , Cloutier, S. and You, F.M. (2016) RGAugury: a pipeline for genome‐wide prediction of resistance gene analogs (RGAs) in plants. BMC Genomics 17, 852. 27806688 10.1186/s12864-016-3197-x PMC5093994

[pbi70192-bib-0052] Liu, C. , Wang, Y. , Peng, J. , Fan, B. , Xu, D. , Wu, J. , Cao, Z. *et al*. (2022a) High‐quality genome assembly and pan‐genome studies facilitate genetic discovery in mung bean and its improvement. Plant Communications 3, 100352. 35752938 10.1016/j.xplc.2022.100352 PMC9700124

[pbi70192-bib-0054] Liu, Y.‐L. , Zheng, L. , Jin, L.‐G. , Liu, Y.‐X. , Kong, Y.‐N. , Wang, Y.‐X. , Yu, T.‐F. *et al*. (2022b) Genome‐wide analysis of the soybean TIFY family and identification of GmTIFY10e and GmTIFY10g response to salt stress. Front. Plant Sci. 13, 845314. 35401633 10.3389/fpls.2022.845314 PMC8984480

[pbi70192-bib-0053] Liu, Y. , Du, H. , Li, P. , Shen, Y. , Peng, H. , Liu, S. , Zhou, G.‐A. *et al*. (2020) Pan‐genome of wild and cultivated soybeans. Cell 182, 162–176.e13. 32553274 10.1016/j.cell.2020.05.023

[pbi70192-bib-0055] Lovell, J.T. , Sreedasyam, A. , Schranz, M.E. , Wilson, M. , Carlson, J.W. , Harkess, A. , Emms, D. *et al*. (2022) GENESPACE tracks regions of interest and gene copy number variation across multiple genomes. eLife 11, e78526. 10.7554/eLife.78526 36083267 PMC9462846

[pbi70192-bib-0056] Manni, M. , Berkeley, M.R. , Seppey, M. , Simão, F.A. and Zdobnov, E.M. (2021) BUSCO update: Novel and streamlined workflows along with broader and deeper phylogenetic coverage for scoring of eukaryotic, prokaryotic, and viral genomes. Mol. Biol. Evol. 38, 4647–4654. 34320186 10.1093/molbev/msab199 PMC8476166

[pbi70192-bib-0057] Mazzucotelli, E. , Belloni, S. , Marone, D. , De Leonardis, A.M. , Guerra, D. , Di Fonzo, N. , Cattivelli, L. *et al*. (2006) The E3 ubiquitin ligase gene family in plants: regulation by degradation. Curr. Genomics 7, 509–522. 18369404 10.2174/138920206779315728 PMC2269001

[pbi70192-bib-0058] Nawrocki, E.P. and Eddy, S.R. (2013) Infernal 1.1: 100‐fold faster RNA homology searches. Bioinformatics 29, 2933–2935. 24008419 10.1093/bioinformatics/btt509 PMC3810854

[pbi70192-bib-0059] Ou, S. , Su, W. , Liao, Y. , Chougule, K. , Ware, D. , Peterson, T. , Jiang, N. *et al*. (2019) Benchmarking transposable element annotation methods for creation of a streamlined, comprehensive pipeline. Genome Biol. 20, 275. 31843001 10.1186/s13059-019-1905-y PMC6913007

[pbi70192-bib-0060] Perez‐Rial, A. , Carmona, A. , Ali, L. , Rubio, J. , Millan, T. , Castro, P. and Die, J.V. (2024) Phenotypic and genetic characterization of a near‐isogenic line pair: insights into flowering time in chickpea. BMC Plant Biol. 24, 709. 39054447 10.1186/s12870-024-05411-y PMC11270784

[pbi70192-bib-0061] Pertea, M. , Pertea, G.M. , Antonescu, C.M. , Chang, T.‐C. , Mendell, J.T. and Salzberg, S.L. (2015) StringTie enables improved reconstruction of a transcriptome from RNA‐seq reads. Nat. Biotechnol. 33, 290–295. 25690850 10.1038/nbt.3122 PMC4643835

[pbi70192-bib-0062] Purushothaman, R. , Upadhyaya, H.D. , Gaur, P.M. , Gowda, C.L.L. and Krishnamurthy, L. (2014) Kabuli and desi chickpeas differ in their requirement for reproductive duration. Field Crop Res. 163, 24–31.

[pbi70192-bib-0063] Qin, P. , Lu, H. , Du, H. , Wang, H. , Chen, W. , Chen, Z. , He, Q. *et al*. (2021) Pan‐genome analysis of 33 genetically diverse rice accessions reveals hidden genomic variations. Cell 184, 3542–3558. 34051138 10.1016/j.cell.2021.04.046

[pbi70192-bib-0064] Ramu, P. , Srivastava, R.K. , Sanyal, A. , Fengler, K. , Cao, J. , Zhang, Y. , Nimkar, M. *et al*. (2023) Improved pearl millet genomes representing the global heterotic pool offer a framework for molecular breeding applications. Communications Biology 6, 902. 37667032 10.1038/s42003-023-05258-3 PMC10477261

[pbi70192-bib-0065] Roorkiwal, M. , Bharadwaj, C. , Barmukh, R. , Dixit, G.P. , Thudi, M. , Gaur, P.M. , Chaturvedi, S.K. *et al*. (2020) Integrating genomics for chickpea improvement: achievements and opportunities. Theor. Appl. Genet. 133, 1703–1720. 32253478 10.1007/s00122-020-03584-2 PMC7214385

[pbi70192-bib-0066] Sedlazeck, F.J. , Rescheneder, P. , Smolka, M. , Fang, H. , Nattestad, M. , von Haeseler, A. and Schatz, M.C. (2018) Accurate detection of complex structural variations using single‐molecule sequencing. Nat. Methods 15, 461–468. 29713083 10.1038/s41592-018-0001-7 PMC5990442

[pbi70192-bib-0067] Shang, L. , Li, X. , He, H. , Yuan, Q. , Song, Y. , Wei, Z. , Lin, H. *et al*. (2022) A super pan‐genomic landscape of rice. Cell Res. 32, 878–896. 35821092 10.1038/s41422-022-00685-z PMC9525306

[pbi70192-bib-0068] Shu, K. and Yang, W. (2017) E3 ubiquitin ligases: Ubiquitous actors in plant development and abiotic stress responses. Plant Cell Physiol. 58, 1461–1476. 28541504 10.1093/pcp/pcx071 PMC5914405

[pbi70192-bib-0069] Singh, P. , Sundaram, K.T. , Vinukonda, V.P. , Venkateshwarlu, C. , Paul, P.J. , Pahi, B. , Gurjar, A. *et al*. (2024) Superior haplotypes of key drought‐responsive genes reveal opportunities for the development of climate‐resilient rice varieties. Communications Biology 7, 89. 38216712 10.1038/s42003-024-05769-7 PMC10786901

[pbi70192-bib-0070] Sinha, P. , Singh, V.K. , Saxena, R.K. , Khan, A.W. , Abbai, R. , Chitikineni, A. , Desai, A. *et al*. (2020) Superior haplotypes for haplotype‐based breeding for drought tolerance in pigeonpea (*Cajanus cajan* L.). Plant Biotechnol. J. 18, 2482–2490. 32455481 10.1111/pbi.13422 PMC7680530

[pbi70192-bib-0071] Stanke, M. , Diekhans, M. , Baertsch, R. and Haussler, D. (2008) Using native and syntenically mapped cDNA alignments to improve de novo gene finding. Bioinformatics 24, 637–644. 18218656 10.1093/bioinformatics/btn013

[pbi70192-bib-0072] Sun, Y. , Qiao, Z. , Muchero, W. and Chen, J.‐G. (2020) Lectin receptor‐like kinases: The sensor and mediator at the plant cell surface. Front. Plant Sci. 11, 596301. 33362827 10.3389/fpls.2020.596301 PMC7758398

[pbi70192-bib-0073] Takagi, H. , Hempton, A.K. and Imaizumi, T. (2023) Photoperiodic flowering in Arabidopsis: Multilayered regulatory mechanisms of CONSTANS and the florigen FLOWERING LOCUS T. Plant Communications 4, 100552. 36681863 10.1016/j.xplc.2023.100552 PMC10203454

[pbi70192-bib-0074] Tarailo‐Graovac, M. and Chen, N. (2009) Using RepeatMasker to identify repetitive elements in genomic sequences. Current Protocols in Bioinformatics, Chapter 4, 4.10.1–4.10.14. 10.1002/0471250953.bi0410s25 19274634

[pbi70192-bib-0075] Thudi, M. , Chitikineni, A. , Liu, X. , He, W. , Roorkiwal, M. , Yang, W. , Jian, J. *et al*. (2016) Recent breeding programs enhanced genetic diversity in both desi and kabuli varieties of chickpea (*Cicer arietinum* L.). Sci. Rep. 6, 38636. 27982107 10.1038/srep38636 PMC5159902

[pbi70192-bib-0076] Torkamaneh, D. , Lemay, M.‐A. and Belzile, F. (2021) The pan‐genome of the cultivated soybean (PanSoy) reveals an extraordinarily conserved gene content. Plant Biotechnol. J. 19, 1852–1862. 33942475 10.1111/pbi.13600 PMC8428833

[pbi70192-bib-0077] van Ooijen, G. , Mayr, G. , Kasiem, M.M.A. , Albrecht, M. , Cornelissen, B.J.C. and Takken, F.L.W. (2008) Structure‐function analysis of the NB‐ARC domain of plant disease resistance proteins. J. Exp. Bot. 59, 1383–1397. 18390848 10.1093/jxb/ern045

[pbi70192-bib-0088] Varshney, R. K. , Thudi, M. , Roorkiwal, M. , He, W. , Upadhyaya, H. D. , Yang, W. *et al*. (2019). Resequencing of 429 chickpea accessions from 45 countries provides insights into genome diversity, domestication and agronomic traits. Nat. Genet. 51, 857–864. 31036963 10.1038/s41588-019-0401-3

[pbi70192-bib-0078] Varshney, R.K. , Roorkiwal, M. , Sun, S. , Bajaj, P. , Chitikineni, A. , Thudi, M. , Singh, N.P. *et al*. (2021) A chickpea genetic variation map based on the sequencing of 3,366 genomes. Nature 599, 622–627. 34759320 10.1038/s41586-021-04066-1 PMC8612933

[pbi70192-bib-0079] Varshney, R.K. , Song, C. , Saxena, R.K. , Azam, S. , Yu, S. , Sharpe, A.G. , Cannon, S. *et al*. (2013) Draft genome sequence of chickpea (*Cicer arietinum*) provides a resource for trait improvement. Nat. Biotechnol. 31, 240–246. 23354103 10.1038/nbt.2491

[pbi70192-bib-0080] Varshney, R.K. , Thudi, M. , Nayak, S.N. , Gaur, P.M. , Kashiwagi, J. , Krishnamurthy, L. , Jaganathan, D. *et al*. (2014) Genetic dissection of drought tolerance in chickpea (*Cicer arietinum* L.). Theor. Appl. Genet. 127, 445–462. 24326458 10.1007/s00122-013-2230-6 PMC3910274

[pbi70192-bib-0081] Wang, O. , Cheng, X. , Drmanac, R. , & Peters, B. A. (2023a). A simple cost‐effective method for whole‐genome sequencing, haplotyping, and assembly. Methods Mol. Biol., 2590, 101–125. 36335495 10.1007/978-1-0716-2819-5_7

[pbi70192-bib-0082] Wang, O. , Chin, R. , Cheng, X. , Wu, M.K.Y. , Mao, Q. , Tang, J. , Sun, Y. *et al*. (2019) Efficient and unique cobarcoding of second‐generation sequencing reads from long DNA molecules enabling cost‐effective and accurate sequencing, haplotyping, and de novo assembly. Genome Res. 29, 798–808. 30940689 10.1101/gr.245126.118 PMC6499310

[pbi70192-bib-0083] Wang, S. , Qian, Y.‐Q. , Zhao, R.‐P. , Chen, L.‐L. and Song, J.‐M. (2023b) Graph‐based pan‐genomes: increased opportunities in plant genomics. J. Exp. Bot. 74, 24–39. 36255144 10.1093/jxb/erac412

[pbi70192-bib-0084] Weisenfeld, N.I. , Kumar, V. , Shah, P. , Church, D.M. and Jaffe, D.B. (2017) Direct determination of diploid genome sequences. Genome Res. 27, 757–767. 28381613 10.1101/gr.214874.116 PMC5411770

[pbi70192-bib-0085] Yang, T. , Liu, R. , Luo, Y. , Hu, S. , Wang, D. , Wang, C. , Pandey, M.K. *et al*. (2022) Improved pea reference genome and pan‐genome highlight genomic features and evolutionary characteristics. Nat. Genet. 54, 1553–1563. 36138232 10.1038/s41588-022-01172-2 PMC9534762

[pbi70192-bib-0086] Yuan, Y. , Bayer, P.E. , Batley, J. and Edwards, D. (2021) Current status of structural variation studies in plants. Plant Biotechnol. J. 19, 2153–2163. 34101329 10.1111/pbi.13646 PMC8541774

[pbi70192-bib-0087] Zhou, Y. , Minio, A. , Massonnet, M. , Solares, E. , Lv, Y. , Beridze, T. , Cantu, D. *et al*. (2019) The population genetics of structural variants in grapevine domestication. Nature Plants 5, 965–979. 31506640 10.1038/s41477-019-0507-8

