# Supplementary material for: An Australian chickpea pan‐genome provides insights into genome organization and offers opportunities for enhancing drought adaptation for crop improvement
Source: Plant Biotechnol J. 2025 Jun 18;23(9):3967–83. doi: 10.1111/pbi.70192 (PMC12392939; doi:10.1111/pbi.70192)
Supplement: Supplementary file 1 — Figure S1 GC content distribution across different Australian chickpea cultivars. Figure S2 Length distribution of various gene features across different chickpea cultivars. Figure S3 Gene Ontology (GO) enrichment analysis of core and dispensable gene families. Figure S4 Genome‐wide distribution of InDels across the Australian chickpea cultivars. [file PBI-23-3967-s001.pdf]

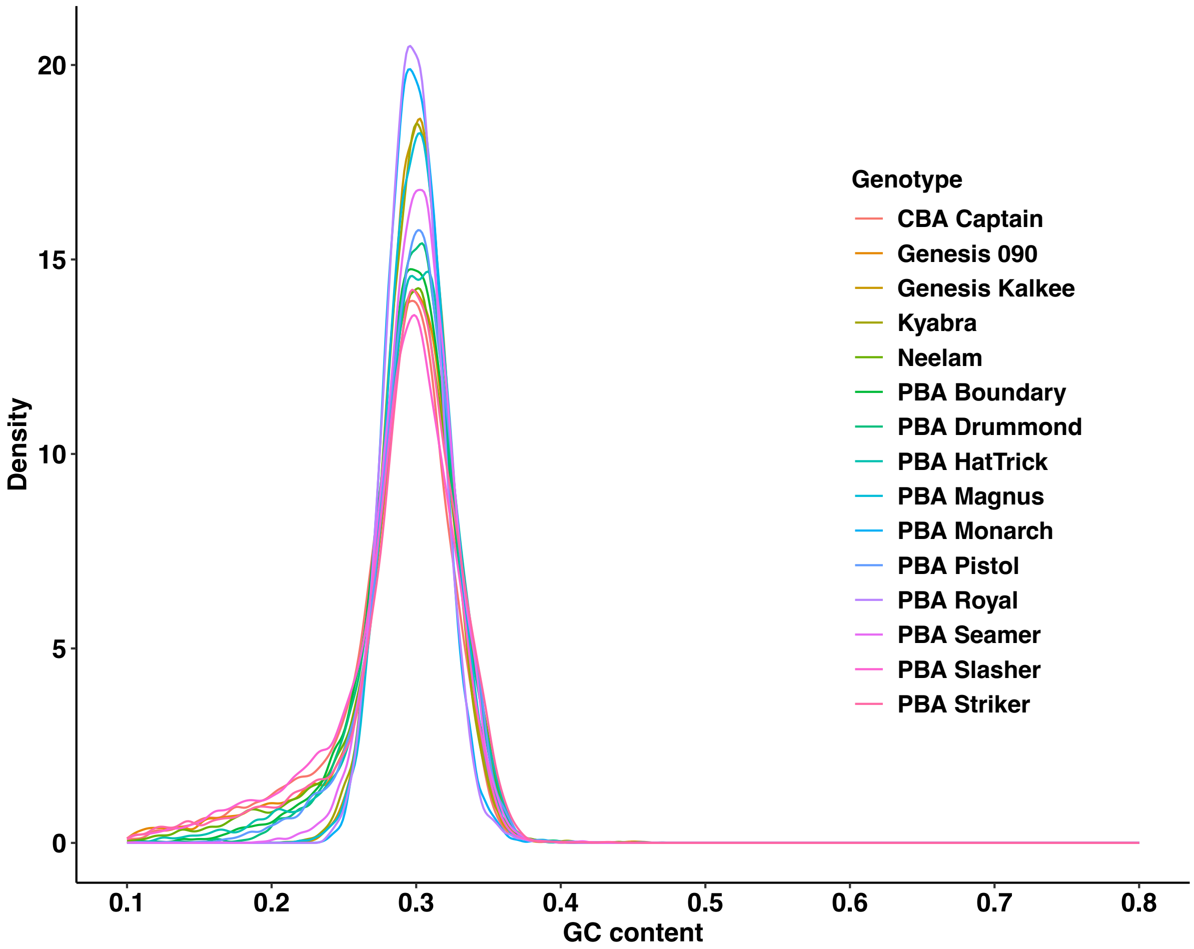

Supplementary Figure 1. GC content distribution across different Australian chickpea cultivars. The GC content was calculated for each genome using 50-kb non-overlapping sliding windows.

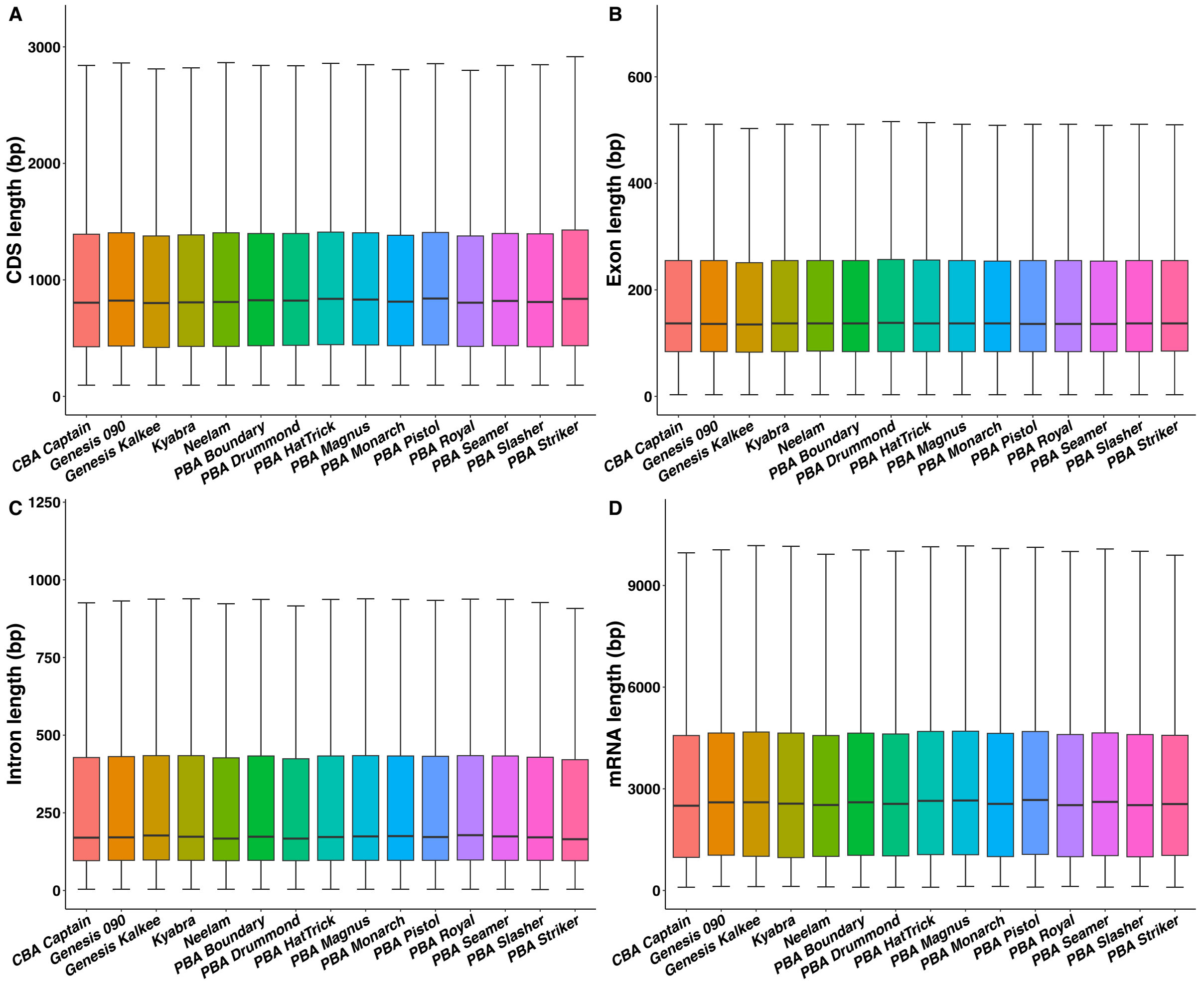

Supplementary Figure 2. Length distribution of various gene features across different chickpea cultivars. The boxplots denote the average CDS (A), exon (B), intron (C), and mRNA (D) lengths. The upper and lower whisker (indicated by vertical lines) represent the 25% and 75% quartiles and the median (as a solid line).

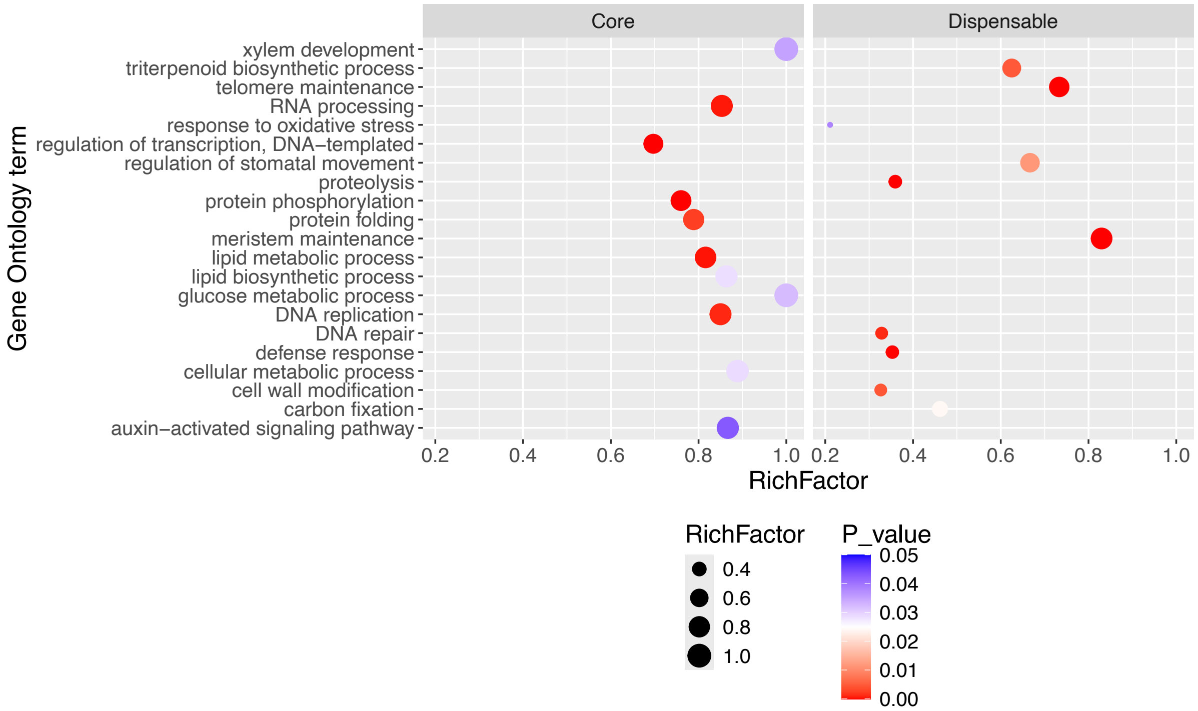

Supplementary Figure 3. Gene Ontology (GO) enrichment analysis of core and dispensable gene families. The bubble plot highlights the significantly enriched GO terms among the core and dispensable gene families across the chickpea cultivars used in this study. The dot size and color correspond to Rich Factor and P-value, respectively. The Rich Factor represents the ratio of the number of genes with the GO term in the core or dispensable gene families and the total number of genes with annotation in the respective GO term.

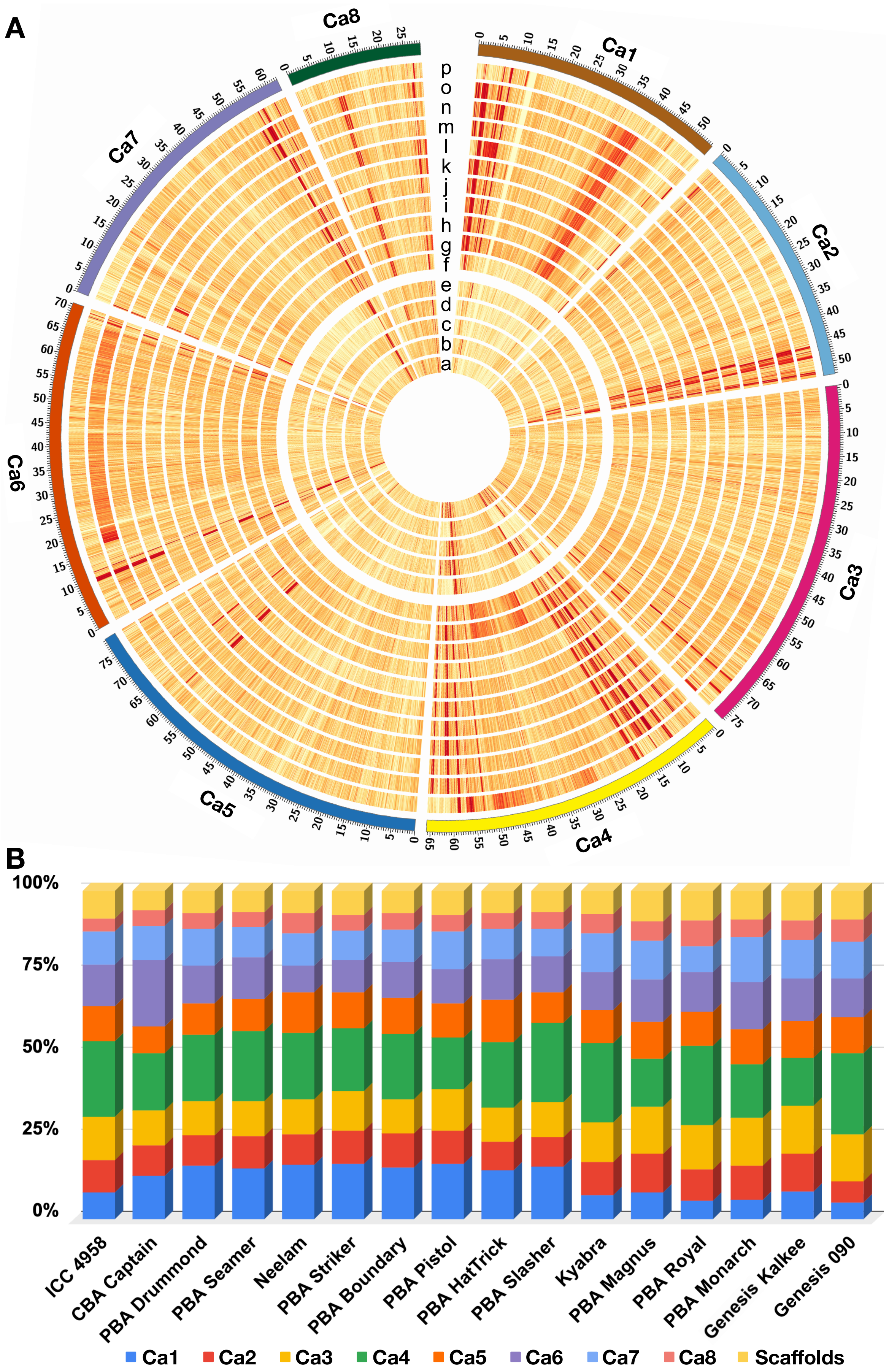

Supplementary Figure 4. Genome-wide distribution of InDels across the Australian chickpea cultivars. A) Circos plot depicting the distribution of InDels found in the Australian

cultivars. Five different tracks (in to out) of the circular plot show the Kabuli varieties ordered by their release year: a, Genesis 090; b, Genesis Kalkee; c, PBA Monarch; d, PBA Royal; e, PBA Magnus. The 11 tracks (in to out) of the circular plot show the Desi varieties ordered by their release year: f, Kyabra; g, PBA Slasher; h, PBA HatTrick; i, PBA Pistol; j, PBA Boundary; k, PBA Striker; l, Neelam; m, PBA Seamer; n, PBA Drummond; o, CBA Captain. The outermost track (p) denotes ICC 4958. The color scale represents the InDel density calculated over 50kb windows, where yellow and red denote low and high density. B) The stacked barplot depicts the chromosome-wise distribution of InDels for each variety.
